# Therapy With Carboplatin and Anti-PD-1 Antibodies Before Surgery Demonstrates Sustainable Anti-Tumor Effects for Secondary Cancers in Mice With Triple-Negative Breast Cancer

**DOI:** 10.3389/fimmu.2020.00366

**Published:** 2020-03-05

**Authors:** Meizhuo Gao, Tie Wang, Litong Ji, Shuping Bai, Lining Tian, Hongjiang Song

**Affiliations:** ^1^Department of General Surgery, Fourth Affiliated Hospital of Harbin Medical University, Harbin, China; ^2^Department of Gastrointestinal Surgery, Harbin Medical University Cancer Hospital, Harbin, China; ^3^Department of Internal Medical Oncology, Harbin Medical University Cancer Hospital, Harbin, China; ^4^Department of Medical Education, First Affiliated Hospital of Harbin Medical University, Harbin, China

**Keywords:** triple-negative breast cancer, carboplatin, immune checkpoint inhibitor, surgery, tumor microenvironment

## Abstract

Patients with triple-negative breast cancer (TNBC) suffer an unfavorable prognosis. Carboplatin (CBDCA) as a cytotoxic reagent has been widely administered to patients with cancer including TNBC. Programmed cell death protein 1 (PD-1) is an immune checkpoint, blockade of which unleashes T cell functions that kill cancer cells. However, the efficacy of CBDCA combined with anti-PD-1 antibodies in TNBC has not been determined. Patient-derived xenografts (PDX) were implanted to immune-deficient mice. Three mouse TNBC cell lines (4T1, EMT6, and E0771) were seeded to immune-competent mice. Tumor volumes and survival rates were monitored. CBDCA and anti-PD-1 antibodies were administered by intra-peritoneal injection at designated time points. Total CD8^+^ T cells, memory CD8^+^ T cells, and CD103^+^ dendritic cells (DC) in the tumor were measured by flow cytometry. Tumor-specific CD8^+^ T cells were quantified by the ELISpot assay. Administration of CBDCA to PDX-bearing mice induced increased levels of tumor cell necrosis and reduced tumor size. Treatment with CBDCA and anti-PD-1 antibodies reduced TNBC tumor volumes and slightly improved survival rates. More importantly, therapy with CBDCA and anti-PD-1 antibodies before surgery showed a remarkably improved, sustainable protection against a secondary tumor after surgery by a CD8^+^- T-cell-dependent manner, which required CCL4 expressed in the tumor and subsequently CD103^+^ DC recruited to the tumor microenvironment. Immunochemotherapy with CBDCA and anti-PD-1 antibodies before surgery improves the outcome of a secondary tumor after surgery via increasing the number of tumor-specific CD8^+^ T cells in the tumor microenvironment of murine TNBC. These results highlight the possibility to utilize this regimen in clinical practice.

## Introduction

Breast cancer is the most common cancer and one of the leading causes of cancer-related mortality in women (1). Among breast cancer, triple-negative breast cancer (TNBC) is a special type, where expression of estrogen receptor, progesterone receptor, and human epidermal growth factor receptor (HER) 2 is lacking (2). Due to resistance to hormonal therapy like tamoxifen and anti-HER2 treatment like trastuzumab, the prognosis of TNBC remains poor as a result of metastases or recurrence (3).

Chemotherapy is usually administered to all TNBC patients with a tumor >5 mm in diameter regardless of the status of axillary nodes (4). As deficiency in DNA damage repair is a signature of TNBC (2, 5), interstrand crosslinking agents that bind DNA like carboplatin (CBDCA) have been tested in clinical trials, where CBDCA shows advantages in improving the rate of pathological complete response (6). Similar benefits of CBDCA have been observed in non-randomized studies (7–10). These clinical data highlight the potential clinical application of CBDCA in TNBC patients. However, a fraction of TNBC patients fail to respond to CBDCA, and some of those with initial response suffer recurrent disease after surgery. Therefore, it is important to introduce a sustainable treatment in TNBC patients.

Immune checkpoint inhibitors have gained a great deal of attention from oncologists as their efficacies in immunogenic tumors by unleashing T cell functions (11). Programmed cell death protein 1 (PD-1) blockade has been tested in clinical settings of TNBC patients (12), but results from this relatively small cohort indicated acceptable safety of anti-PD-1 therapy and a minority of involved patients gained disease amelioration benefits. More importantly, the above-mentioned study (12) has shed light on the possibility of treating TNBC patients with anti-PD-1 therapy, probably combined with other therapeutic options, since pre-clinical trials have shown no survival benefit in TNBC-bearing mice treated with solo anti-PD-1 antibodies, although anti-PD-1 antibodies combined with an oncolytic virus indicated survival benefit in the same study (13).

The combination of CBDCA and anti-PD-1 antibodies has been utilized as immunochemotherapy in small trials including squamous non-small-cell lung cancer (14, 15), ovarian cancer (16), and others. However, it still remains unclear whether the combination of CBDCA and anti-PD-1 antibodies can induce sustainable responses in TNBC patients. In the current study, we tested the hypothesis that the combination of CBDCA and anti-PD-1 antibodies is efficient for experimental TNBC. Here, we report that the combination of CBDCA and anti-PD-1 antibodies improves survival rates as neoadjuvant immunochemotherapy for secondary tumors after surgery in three murine TNBC models, and enhance tumor-infiltrating T cell functions by elevating CCL4 expression and increasing the number of CD103^+^ dendritic cells (DC) in the tumor microenvironment.

## Materials and Methods

### Ethics Consideration

The ethics committee of Forth Affiliated Hospital of Harbin Medical University reviewed, approved, and supervised this proposal (approval number: 20150017). The patients involved in this study agreed to use their tumor cells and signed the written consent forms.

### Patient-Derived Xenografts (PDX)

TNBC was surgically removed from four TNBC patients (Patient 201600787, 39 years old; Patient 201821212, 52 years old; Patient 201821344, 65 years old: Patient 201711434, 53 years old) with a stage 3A tumor who received the diagnosis by assessments of histology and immunohistochemistry. The implantation procedure to NOD/SCID (non-obese diabetic/severe combined immunodeficient) mice (purchased from the Jackson Laboratory, Bar Harbor, ME, USA) followed a previous report (17). Briefly, a small fraction of fresh tumor (up to 8 mm^3^) was implanted into the second left mammary fat pads of female NOD-SCID female mice (3–4 weeks old).

### Cell Lines and Cell Culture

Murine TNBC cell lines (4T1, EMT6, and E0771) were purchased from American Type Culture Collection (Manassas, VA, USA). Cells were cultured in Dulbecco's Modified Eagle's Medium (Thermo Fisher Scientific, Waltham, MA, USA) supplemented with 10% fetal bovine serum (FBS, purchased from Thermo Fisher Scientific) and maintained at 37°C with 5% CO_2_.

### Orthotopically Planted Tumor Models

All mice were purchased from the Jackson Laboratory (Bar Harbor, ME, USA). Female mice (6 to 8 weeks old) were utilized in the TNBC models. Balb/c mice were used for the 4T1 and EMT6 murine TNBC models, whereas C57/Bl6 mice were used for the E0771 model. Briefly, 2 × 10^5^ 4T1 or EMT6 tumor cells were injected into the second left mammary fat pad. A total of 1 × 10^6^ E0771 tumor cells were implanted into the second left mammary fat pad. Tumor volume was measured twice a week using an electronic caliper, which was calculated as (π × length ×width^2^)/6. When tumor volume reached 1,500 mm^3^, mice were sacrificed.

At day 20 after the primary tumor implantation, tumors were surgically removed along with the skin associated with the tumor (13). Four days after surgery, the same amount of tumor cells as described above in each model were seeded in the second right mammary fat pad (referred to as the secondary tumor implantation).

### Anti-cancer Treatment

CBDCA was purchased from Cayman Chemical Company (Ann Arbor, MI, USA). Mice received a dose of 100 mg/kg of CBDCA by intra-peritoneal injection at designated time points (18).

Anti-PD-1 antibodies (clone: RMP1-14) were purchased from Bio X cell (West Lebanon, NH, USA). A dose of 100 μg/mouse of anti-PD-1 antibodies was administered at designated time points intra-peritoneally as described before (13). Mice treated with the same dose of isotype control antibodies (rat IgG2a, Bio X cell) were utilized as control.

### Neutralizing Antibodies

For depleting CD8^+^ T cells, anti-CD8 (clone 2.43; Bio X cell) was administered with a dose of 250 μg in 100 μl of PBS by intra-peritoneal injection at day 7 after the primary tumor implantation (19). At days 10, 14, 21, 24, and 28, another dose of 100 μg of antibodies was applied intra-peritoneally. Control mice received isotype control antibodies (rat anti-mouse IgG2b, Bio X cell) with the same regimen as CD8^+^ T cell depletion.

For neutralizing CCL4, anti-mouse CCL4 antibodies (polyclonal, purchased from R&D Systems, Minneapolis, MN, USA) were administered to mice with a dose of 50 μg in 100 μl of PBS per mouse by intra-peritoneal injection at days 7, 10, 14, 21, 24, and 28 (20). The corresponding isotype control antibodies (normal goat IgG control, R&D Systems) were used in control mice.

### Immunofluorescence

Frozen sections of resected tumors were cut into 4- to 6-μm serial slices. Slices were fixed in 1% of formalin for 30 min at room temperature, followed by blocking with 10% goat serum (Thermo Fisher Scientific). Samples subsequently stained with anti-CD103-AF488 (1:100, Thermo Fisher Scientific) and anti-CCL4-AF647 (1:100, purchased from Abcam, Cambridge, MA, USA) antibodies for 30 min at the room temperature. Receptor-interacting serine/threonine-protein kinase (RIP) 3 antibodies (PA5-19956, Thermo Fisher Scientific) were used to stain apoptotic cells with a titration of 1:100 by immunofluorescence (21).

### Enzyme-Linked Immunospot (ELISpot)

ELISpot plates (96-well) were pre-coated with anti-mouse interferon (IFN) γ monoclonal antibody overnight at 4°C following the manufacturer's instructions (BD Biosciences, San Jose, CA, USA). Subsequently, plates were blocked with 10% FBS for 2 h at room temperature. By using a mouse CD8^+^ T cell isolation kit (Miltenyi Biotec, Bergisch Gladbach, Germany), tumor-infiltrating CD8^+^ T cells were isolated from single-cell suspensions that were achieved by a mouse tumor dissociation kit (Miltenyi Biotec) following the manufacturer's instruction (22). Purified CD8^+^ T cells were seeded into the above-mentioned ELISpot plates (10^5^ cells/well) and stimulated overnight with or without live tumor cells according to the implanted cell line (2 × 10^4^ per well). Plates were washed twice with ddH_2_O, and then incubated with biotinylated detection antibody for 2 h at room temperature. After thorough washes, plates were incubated with streptavidin-HRP. Spots were developed by using the AEC substrate Reagent Set (BD Bioscience) and counted under a dissecting microscope.

### Flow Cytometry

Single-cell suspensions from tumors were achieved by enzyme digestion and mechanical dissociation using a commercial kit (Miltenyi Biotec) (22). Briefly, tumors were removed from mice when indicated and minced into small pieces of 2–4 mm in diameter. Tumor pieces were then digested by enzymes provided in the kit for 40 min after dissociation for 1 min. Following digestion, tissues underwent another round of dissociation. Single cell suspensions were achieved by filtering samples through 70-μm strainers and centrifuged (300× g for 10 min at 4°C).

For cell death assessments, a Dead Cell Apoptosis Kit with Annexin V Alexa Fluor 488 & Propidium Iodide (PI) (Thermo Fisher Scientific) was used for flow cytometry analyses according to the manufacturer's guides.

Tumor cells were then incubated with Fixable Viability Dye eFluro 780 (1:1000 in PBS, eBioscience/Thermo Fisher Scientific) for 30 min at 4°C. Subsequently, cells were blocked with FC blocker (1:100, eBiosience) for 10 min at room temperature. Samples then were incubated with antibodies with designated titrations (listed in Table 1). Cells were applied to an Attune NxT flow cytometer (Thermo Fisher Scientific). Data were analyzed by software Kaluza 2.1 (Beckman Coulter, Brea, CA, USA).

**Table 1:** Antibodies used for flow cytometry analyses.

**Antibody-chrome (clone)**	**Manufacturer**	**Titration**
CD3e-PE-Cy7 (145-2C11)	eBioscience	1:100
CD4-FITC (RM4-5)	eBioscience	1:100
CD8a-PerCP-Cy5.5 (53-6.7)	eBioscience	1:100
CD62L-PE (MEL-14)	eBioscience	1:100
CD44-APC (IM7)	eBioscience	1:100
CD45-APC (30-F11)	eBioscience	1:100
MHCII-SuperBright 702(M5/114.15.2)	eBioscience	1:100
CD11b-PE-Cy7 (M1/70)	eBioscience	1:100
CD11c-PE-Cy5.5 (N418)	eBioscience	1:100
CD103-FITC (2E7)	eBioscience	1:100
CCL4-PE (ab45690)	Abcam	1:100

### Statistical Analysis

Numeric data were presented as the mean ± standard deviation (SD), and were analyzed by Student *t-*tests (for two groups), one-way ANOVA (for more than two groups at one time points) with Bonferroni *post-hoc* tests, or two-way ANOVA (for more than two groups at multiple time points) with Tukey's *post-hoc* tests. Survivals were presented by the Kaplan-Meier method and compared by the log-rank test. Contingency data were compared by Chi-square tests. A two-tailed *p*-value of <0.05 was considered as statistical significance. Statistical analyses were performed using GraphPad Prism 7 for Windows (GraphPad Software, San Diego, CA, USA).

## Results

### CBDCA Effectively Induces Necrosis in Patient-Derived Xenografts of TNBC Tumors

**Figure 1:**
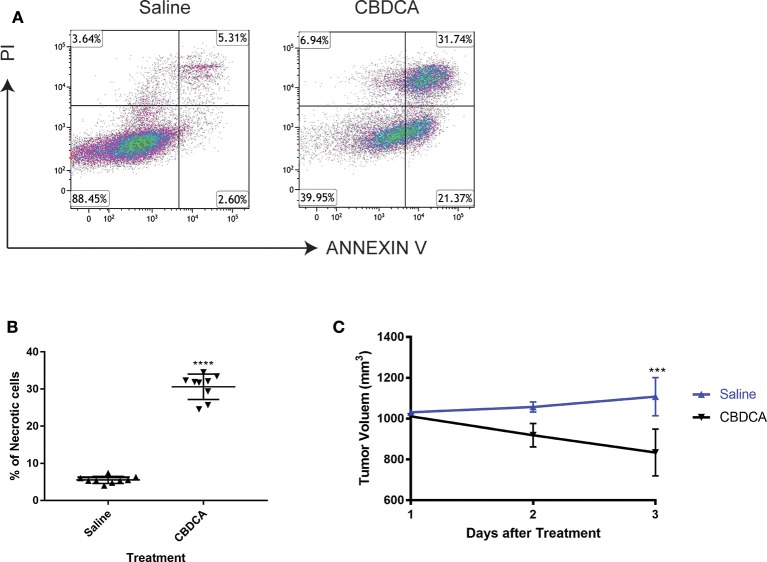
Administration of CBDCA induces xenograft TNBC tumor cell necrosis and reduces tumor volume. TNBC cells were collected and planted to NOD/SCID mice as described. Mice were treated with CBDCA, or saline as controls, when the tumor size reached 1,000 mm^3^. Tumors were collected 72 h after treatment. CD45^−^ cells were analyzed for necrosis by staining PI and ANNEXIN V. **(A)** Representative images of flow cytometry data. **(B)** Treatment of CBDCA increased levels of necrotic cells. Tumor cells were collected from Patient 201600787. ****p < 0.0001, by student *t-*tests. *n* = 9 per group. **(C)** Administration of CBDCA reduced tumor volume over time. ***p < 0.001, by student *t*-tests at day 3 after treatment. Data were from the same experiment of **(B)**. CBDCA, carboplatin.

In order to test the efficacy of CBDCA in PDX of TNBC, we initially collected the surgically resected tumor from a TNBC patient (case number: 201600787, 39 years old). TNBC cells were implanted to NOD/SCID mice as previously described (17). When reached a volume of 1,000 mm^3^ (24 days), mice were intra-peritoneally treated with CBDCA (100 mg/kg), or saline (100 μl) as controls. Seventy-two hours later, tumors were removed and analyzed by flow cytometry for necrosis. CD45^−^ cells were pregated as tumor cells, since it has been reported that implanted tumor cells are CD45-negative (17). As shown in Figure 1, CBDCA administration increased levels of necrosis (CBDCA vs. saline, 30.63 ± 3.43% vs. 5.553 ± 0.966%; *p* < 0.0001). We also measured tumor volumes during treatment, whose results were parallel to the degrees of tumor cell necrosis. Shown in Figure 1C, treatment of CBDCA shrank the tumor volume significantly during the 3-day period (*p* < 0.001). Similar results were obtained from three additional patients using the same approach (Supplemental Figure 1). These data indicate that CBDCA causes tumor cell death in the PDX model of TNBC.

### The Combination of CBDCA and Anti-PD-1 Antibodies Is Effective in Killing Experimental TNBC in Mice

**Figure 2:**
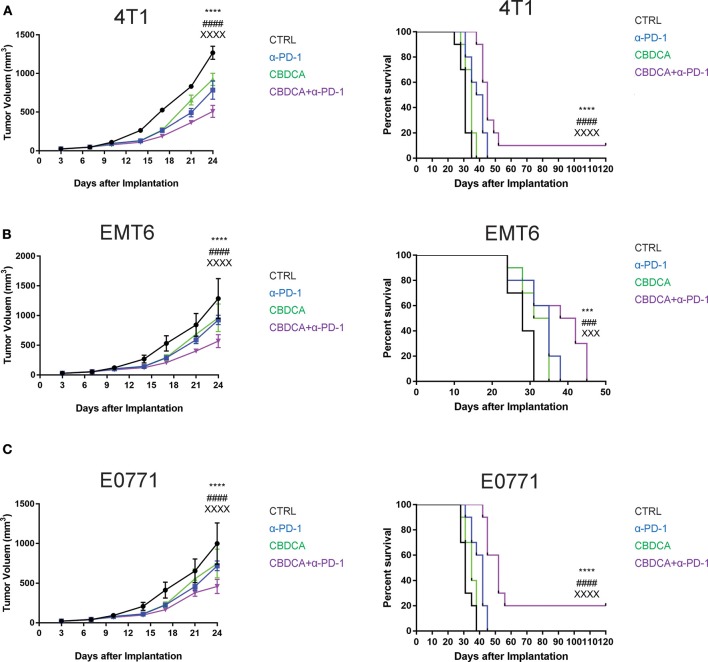
The combination of CBDCA and anti-PD-1 antibodies improves the outcome of murine TNB models. TNBC (4T1, EMT6, and E0771) models were induced as described in Methods. Tumor-bearing mice were treated with CBDCA, anti-PD-1 antibodies, the combination, or saline and isotype control antibodies for anti-PD-1 antibodies as control (CTRL). The combined therapy reduces the tumor size and prolongs survivals in murine TNBC models−4T1 **(A)**, EMT6 **(B)**, and E0771 **(C)**. *n* = 10 in each group from one of triplicated experiments. The symbol “*” denotes the difference between CTRL and CBDCA+α-PD-1. The symbol “#” denotes the difference between α-PD-1 and CBDCA+α-PD-1. The symbol “X” denotes the difference between CBDCA and CBDCA+α-PD-1. ^****, *####, XXXX*^*p* < 0.0001; ^***, *###, XXX*^*p* < 0.001, by two-way ANOVA with Tukey's *post-hoc* tests for the tumor size or log-rank tests for the survival.

Given that the PDX model cannot be used to test the efficacy of anti-cancer immunotherapy like anti-PD-1 treatment, we performed experiments using three syngeneic murine TNBC models-4T1, EMT6, and E0771, as previously described (13, 23–25). These three tumor models share a common feature where spontaneous metastases to the liver, lungs, brain, and bones occur, mimicking advanced stages of breast cancer in human patients. At Day 7 after implantation, tumor-bearing mice were treated by intra-peritoneal injection with CBDCA (100 mg/kg, every 7 days for 2 times), anti-PD-1 antibodies (100 μg/mouse, every second day for 5 times), the combination of both, or saline plus isotype antibodies as controls (CTRL). As demonstrated in Figure 2, in all three models, the combination of CBDCA and anti-PD-1 antibodies reduced the tumor size at the early phase after implantation (CBDCA+α-PD-1 vs. CTRL, *p* < 0.0001) and prolonged the survival of tumor-bearing mice (CBDCA+α-PD-1 vs. CTRL, *p* < 0.0001 in 4T1 and E0771 tumors, and *p* < 0.001 in EMT6 tumor). Although treatment with CBDCA or anti-PD-1 antibodies alone reduced tumor size at Day 24 after implantation (Supplemental Tables 1–3), these two strategies had no therapeutic effects in prolonging the survival (Supplemental Tables 4–6). However, the combination therapy showed efficacies in reducing tumor size and prolonging survival compared to those treated with CBDCA or anti-PD-1 antibodies alone. These data indicate the combination of CBDCA and anti-PD-1 antibodies presents anti-tumor effects in experimental TNBC models.

### Therapy With CBDCA and Anti-PD-1 Antibodies Before Surgery Has a Sustainable Anti-cancer Effect for Secondary Tumors

**Figure 3:**
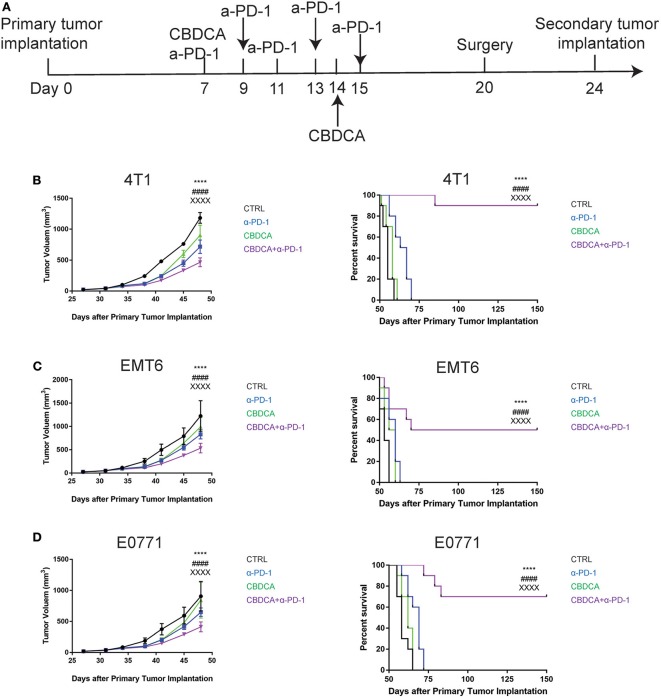
Neoadjuvant therapy with CBDCA and anti-PD-1 antibodies has a sustainable anti-cancer effect in mouse TNBC models. **(A)** The schematic representation of therapeutic schedules of neoadjuvant therapy and surgery. Neoadjuvant therapy of CBDCA and anti-PD-1 antibodies reduces tumor size and improves survival rates of mice bearing secondary tumors of 4T1 **(B)**, EMT6 **(C)**, and E0771 **(D)**. *n* = 10 in each group from one of triplicated experiments. The symbol “*” denotes the difference between CTRL and CBDCA+α-PD-1. The symbol “#” denotes the difference between α-PD-1 and CBDCA+α-PD-1. The symbol “X” denotes the difference between CBDCA and CBDCA+α-PD-1. ^****, *####, XXXX*^*p* < 0.0001, by two-way ANOVA with Tukey's *post-hoc* tests for the tumor size or log-rank tests for the survival.

As shown in Figure 2, although administration of the combination therapy induced necrosis measured by RIP3 immunostaining (21) (Supplemental Figure 2) in implanted TNBC and reduced tumor volume, the long-term survival of tumor-bearing mice treated with the combination of CBDCA and anti-PD-1 antibodies still remained suboptimal due to metastases to other organs, among which the major target organ was the lungs (metastasis patterns were illustrated in Supplemental Figure 3). Consequently, we questioned whether this combination could improve the outcome of surgically treated tumor-bearing mice by preventing metastases or relapse. In order to test the efficacy of the combination of CBDCA and anti-PD-1 antibodies in this context, we utilized a surgical model for TNBC. At Day 20 after primary implantation (3 days after the last administration, Figure 3A), the primary tumor was surgically removed, and 4 days later a secondary tumor was implanted into the second right mammary fat pad, which was left untreated. As shown in Figures 3B–D, in all three TNBC models, combined neoadjuvant therapy of CBDCA and anti-PD-1 significantly reduced tumor volume of secondary breast cancers (CBDCA+α-PD-1 vs. CTRL, *p* < 0.0001), and consequently remarkably improved the survival rate (CBDCA+α-PD-1 vs. CTRL, *p* < 0.0001). Treatment with single-agent (CBDCA or anti-PD-1 antibodies) temporarily reduced tumor size at Day 48 (Supplemental Tables 7–9), but could not prolong the survival (Supplemental Tables 10–12). The combination therapy showed superior anti-tumor effects with respect to CBDCA or anti-PD-1 antibodies. More specifically, tumor-bearing mice treated neoadjuvant therapy of CBDCA combined with anti-PD-1 antibodies showed a survival rate between 50 and 90% across all three models, which were significantly higher than those treated with single-drug regimens or CTRL (Supplemental Tables 10–12, *p* < 0.0001). These data indicate that neoadjuvant therapy of CBDCA and anti-PD-1 antibodies is effective for killing a secondary tumor with a sustainable tumor control.

### Efficacy of Therapy of CBDCA and Anti-PD-1 Antibodies Before Surgery Is Dependent on CD8^+^ T Cells

**Figure 4:**
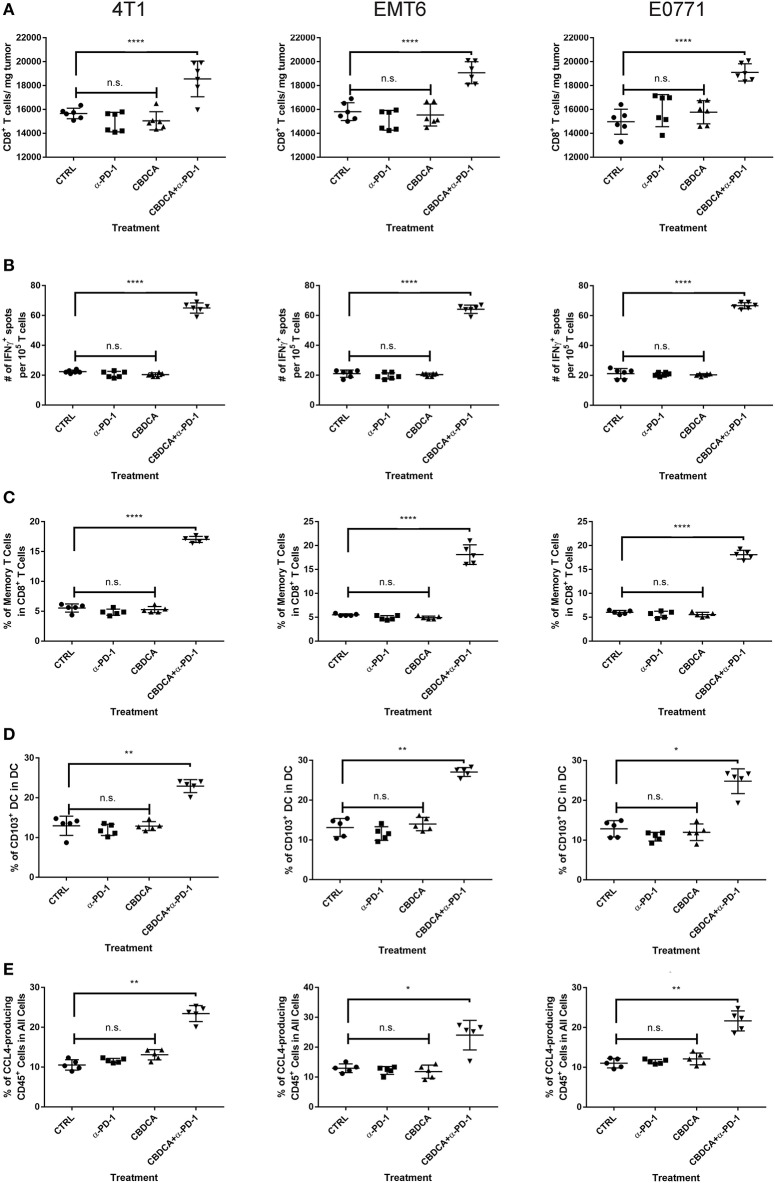
Major immunological parameters in the tumor microenvironment of the secondary tumor. The primary and secondary tumor implantation, as well as treatment were conducted as the schematic shown in Figure 3. At Day 35 after the primary tumor implantation, tumors were collected for flow cytometry analysis. Left columns: 4T1; middle columns: EMT6; right columns: E0771. **(A)** The number of CD8^+^ tumor infiltrating T cells. **(B)** ELISpot assay. **(C)** The percentage of memory CD8^+^ T cells in the tumor. **(D)** The percentage of CD103^+^ DC in the tumor. **(E)** The percentage of CCL4-producing CD45^−^ cells in the tumor. *n* = 5–6 mice/group from one experiment. *p < 0.05 (CBDCA+α-PD-1 vs. CBDCA, α-PD-1, or CTRL); **p < 0.01 (CBDCA+α-PD-1 vs. CBDCA, α-PD-1, or CTRL); ****p < 0.0001 (CBDCA+α-PD-1 vs. CBDCA, α-PD-1, or CTRL); n.s., no significance (CBDCA vs. α-PD-1 vs. CTRL), by one-way ANOVA with Bonferroni *post-hoc* tests.

**Figure 5:**
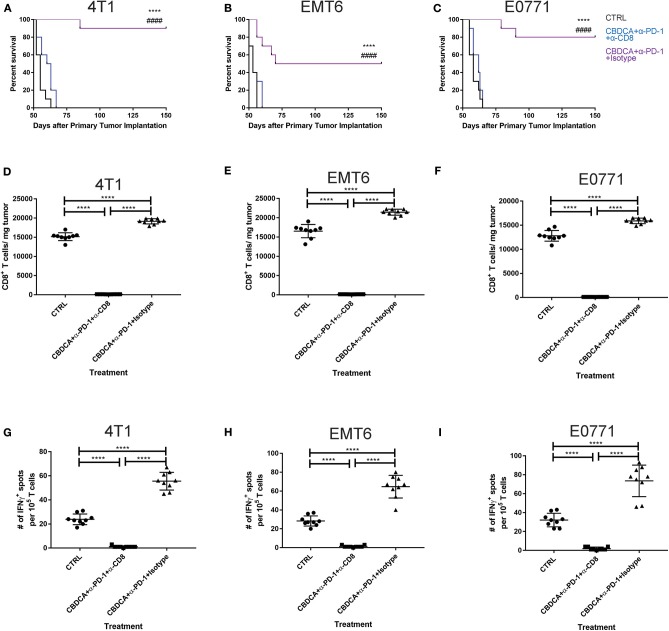
The anti-tumor effect of CBDCA and anti-PD-1 antibodies as neoadjuvant therapy in TNBC models is CD8^+^ T cell-dependent. Neoadjuvant treatment was applied as illustrated in Figure 3. **(A)** Anti-CD8 neutralizing antibodies were administered as described. At Day 35 after the primary tumor implantation, tumors were collected for data analysis. **(A–C)** anti-CD8 neutralizing antibody administration impaired survival rates of tumor-bearing mice treated with CBDCA and anti-PD-1 antibodies. The symbol “*” denotes the difference between CBDCA+α-PD-1+Isotype and CTRL. The symbol “#” denotes the difference between CBDCA+α-PD-1+Isotype and CBDCA+α-PD-1+α-CD8. ^****, *####*^*p* < 0.0001 by log-rank tests, *n* = 10 per group from one of triplicated experiments. **(D–F)** anti-CD8 neutralizing antibody treatment reduced the level of tumor-infiltrating CD8^+^ cells. **(G–I)** anti-CD8 neutralizing antibody supplement decreased the abundance of tumor-specific IFNγ-producing CD8^+^ T cells measured by ELISpot. D-I, by one-way ANOVA with Bonferroni *post-hoc* tests, *n* = 9 per group from one of triplicated experiments. ****p < 0.0001.

Anti-PD-1 antibodies induce CD8^+^ T cell-mediated tumor killing by unleashing T cell functions (11). It has been reported that TNBC patients who had a dominant lymphocytic infiltration showed an improved outcome (26). Therefore, we asked whether the efficacy of neoadjuvant therapy of CBDCA combined with anti-PD-1 antibodies would require intact CD8^+^ T cell functions. Given the treatment benefits were not seen in groups treated with single agents, as well as we only observed significant changes in the abundance of CD8^+^ tumor infiltrating T cells and antigen-specific CD8^+^ T cells in mice treated with CBDCA and anti-PD-1 antibodies (Figures 4A,B), we therefore focused on the tumor killing mechanism of CBDCA combined with anti-PD-1 antibodies. To explore the roles of CD8^+^ T cells in this context, neutralizing anti-CD8 antibodies were delivered intra-peritoneally in the neoadjuvant therapy model described in Figure 3A. The secondary tumor was monitored. Unsurprisingly, the anti-tumor effect of the combination of CBDCA and anti-PD-1 antibodies was abolished in mice with CD8^+^ T cell depletion (Figures 5A–C). With respect to the survival results, at Day 35 after the primary tumor implantation, anti-CD8 neutralizing antibody treatment reduced the abundance of tumor-infiltrating CD8^+^ T cells, which were observed to be elevated in mice treated with CBDCA and anti-PD-1 antibodies (Figures 5D–F). More importantly, CD8^+^ T cell depletion also abolished increased numbers of IFN-γ-producing tumor-specific T cells seen in mice treated with CBDCA and anti-PD-1 antibodies measured by ELISpot at Day 35 after the primary tumor implantation (Figures 5G–I). These data indicate that the combination of CBDCA and anti-PD-1 increases the abundance of tumor infiltrating CD8^+^ T cells and tumor-specific CD8^+^ T cells in the secondary tumors in a setting of immunochemotherapy before surgery for TNBC models.

### Immunochemotherapy Increases the Frequencies of Memory CD8^+^ T Cells and CD103^+^ DC in the Secondary Tumors

**Figure 6:**
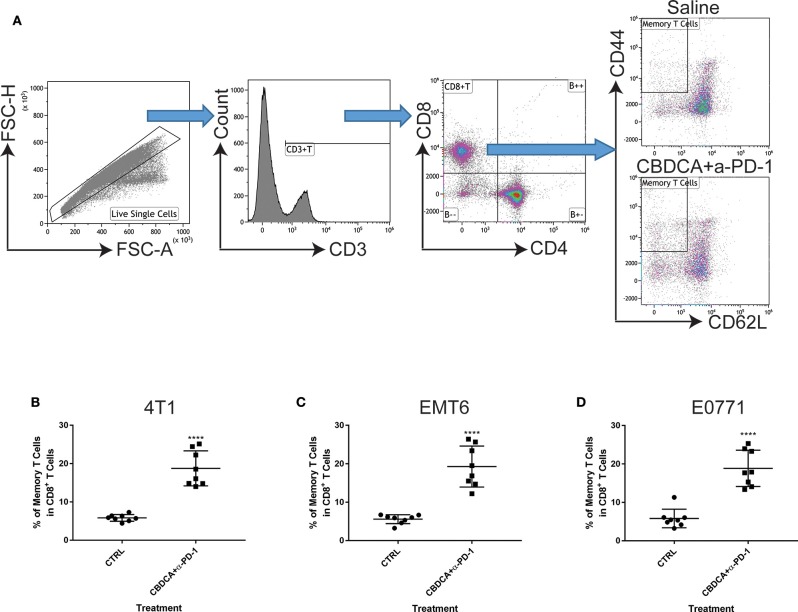
Neoadjuvant therapy with CBDCA and anti-PD-1 antibodies increases the frequencies of memory CD8^+^ T cells in secondary breast cancers. Secondary tumors were collected for flow cytometry analysis at Day 35 after the primary tumor implantation. **(A)** Gating strategies for memory CD8^+^ T cells, which were defined as CD3^+^CD8^+^CD4^−^CD62L^−^CD44^high^ cells. Treatment of CBDCA combined anti-PD-1 antibodies elevated memory CD8^+^ T cells in 4T1 **(B)**, EMT6 **(C)**, and E0771 **(D)** tumors. *n* = 8 per group from one of triplicated experiments. ****p < 0.0001, by two-tail student *t*-tests.

Next, we asked whether the combined neoadjuvant treatment increased the number of memory CD8^+^ T cells in the tumor microenvironment of the secondary breast cancer. At Day 35 after the primary implantation, the secondary tumors were collected for analyzing the abundance of memory CD8^+^ T cells in the tumor microenvironment. As shown in Figure 6, neoadjuvant therapy of CBDCA and anti-PD-1 antibodies increased the number of memory CD8^+^ T cells through all three TNBC murine models (*p* < 0.0001). It is worth noting that the single-regimen treatment like anti-PD-1 antibodies or CBDCA did not change the percentage of memory CD8^+^ T cells in the tumor at this time point and that the combination therapy significantly increased the abundance of memory CD8^+^ T cells compared to treatment with CBDCA or anti-PD-1 (Figure 4C), indicating the possible tumor killing effects executed by memory CD8^+^ T cells. More importantly, the abundance of memory CD8^+^ T cells was negatively correlated with the tumor burden at Day 35 (Supplemental Figure 4) through three TNBC models. These data indicate that memory CD8^+^ T cells that were accumulated in the tumor treated with CBDCA combined with anti-PD-1 antibodies might play a direct killing role in anti-cancer immune responses.

### Combination Therapy Elevates Levels of CD103^+^ DC and CCL4-Producing Tumor Cells in the Tumor Microenvironment

**Figure 7:**
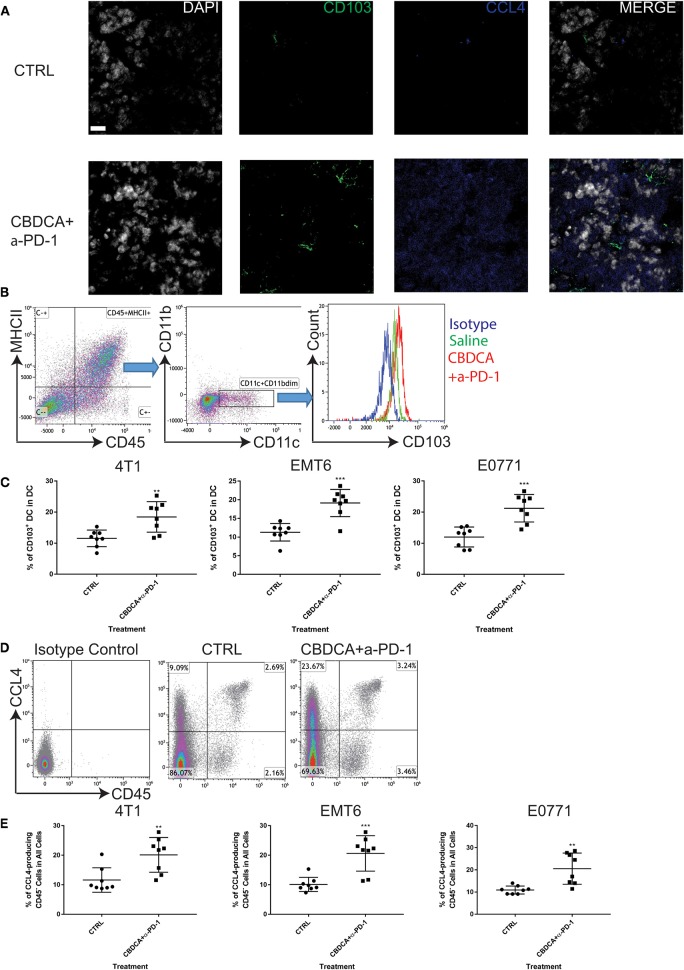
Neoadjuvant therapy of CBDCA and anti-PD-1 antibodies increases the frequencies of CD103^+^ dendritic cells (DC) and CCL4-producing tumor cells in secondary breast cancers. Secondary tumors were collected at Day 35 after the primary tumor implantation. **(A)** Immunofluorescence staining of CD103 and CCL4 in tumors. Scale bar: 9 μm. **(B)** Gating strategies for DC, which were defined as CD45^+^MHCII^+^CD11c^+^CD11b^dim^ cells. **(C)** Treatment of CBDCA plus anti-PD-1 antibodies as a neoadjuvant therapeutic regimen increased the ratio of CD103^+^ DC in implanted TNBC tumors. *n* = 8 per group from one of triplicated experiments. **(D)** Flow cytometry analysis of CCL4-producing cells. **(E)** Treatment of CBDCA and anti-PD-1 antibodies increased the proportion of CD45^−^CCL4^+^ cells in implanted TNBC tumors. *n* = 8 per group from one of triplicated experiments. **p < 0.01; ***p < 0.001, by two-tail student *t*-tests.

Subsequently, we determined the abundance of CD103^+^ DC in the tumor, which are important for the priming of tumor-specific T cells and recruiting tumor-specific memory T cells (27, 28). It has been reported that CCL4 secreted by cancer cells is critical for the recruitment of CD103^+^ DC in the tumor microenvironment, and the further anti-tumor effect (27, 28). Therefore, we measured the expression of CD103 and CCL4 in the tumor microenvironment with or without combination therapy by immunofluorescence. As shown in Figure 7A, at Day 35 after the initial tumor implantation, expression of CD103 and CCL4 was increased in secondary tumors (4T1) treated with CBDCA and anti-PD-1 antibodies.

Next, we measured the abundance of CD103^+^ DC and CCL4-expressing cells by flow cytometry. We demonstrated that treatment with CBDCA and anti-PD-1 before surgery increased the ratio of CD103^+^ DC in total DC in the secondary 4T1 breast tumors by ~1.5-folds (Figures 7B,C, *p* < 0.01) at Day 35 after the primary tumor implantation. Similar results were observed in the other two breast cancer models (Figure 7C). The increases of CD103^+^ DC could only be observed in those treated with CBDCA and anti-PD-1 antibodies, but not mice treated with either reagent (CBDCA+α-PD-1 vs. CBDCA or α-PD-1 or CTRL, *p* < 0.0001, Figure 4D). When calculated as proportions in the total CD45^+^ cells, that of CD103^+^ DC was also increased in those treated with CBDCA and anti-PD-1 antibodies (Supplemental Figure 5). Moreover, the number of CD103^+^ DC in the tumor microenvironment was negatively correlated with the tumor burden (Supplemental Figure 6).

We subsequently measured the number of CCL4-expressing cells. First, we measured the expression and cellular source of CCL4. Single cell suspensions from secondary tumors at Day 35 after the primary tumor implantation were used for flow cytometry analysis. As shown in Figure 7D, the major cellular source of CCL4 in tumors was CD45-negative cells—presumably cancer cells themselves. More importantly, the percentage of CCL4-producing CD45-negative cells was elevated in the secondary 4T1 tumors treated with CBDCA and anti-PD-1 antibodies by ~2-folds (Figure 7E, *p* < 0.01 and Figure 4E), as well as the other two tumor models. Similarly to CD103^+^ DC, the number of CCL4-expressing tumor cells was negatively associated with the tumor burden in the secondary tumor at Day 35 (Supplemental Figure 7).

It is possible that CCL4-producing CD45^+^ leukocytes also play a role in CD103^+^ DC recruitment into the tumor in this context, although leukocytes are not a majority of the cellular source of CCL4. Statistically, however, we did not observe any difference in the abundance of CD45^+^CCL4^+^ induced by treatment across three models (Supplemental Figure 8). More importantly, the abundance of CD45^+^CCL4^+^ was not correlated to the tumor burden of the secondary tumor at Day 35 (Supplemental Figure 9). Thus, these data indicate that CCL4-expressing leukocytes as a minority cellular source of CCL4 have little (if any) roles in the tumor microenvironment.

Altogether, CD103^+^ DC and CCL4-producing tumor cells are enriched in TNBC treated with CBDCA and anti-PD-1 antibodies.

### CCL4 Produced in the Tumor Is Accountable for CD103^+^ DC Recruitment and Subsequent CD8^+^ T Cell Infiltration in the Tumor Microenvironment

**Figure 8:**
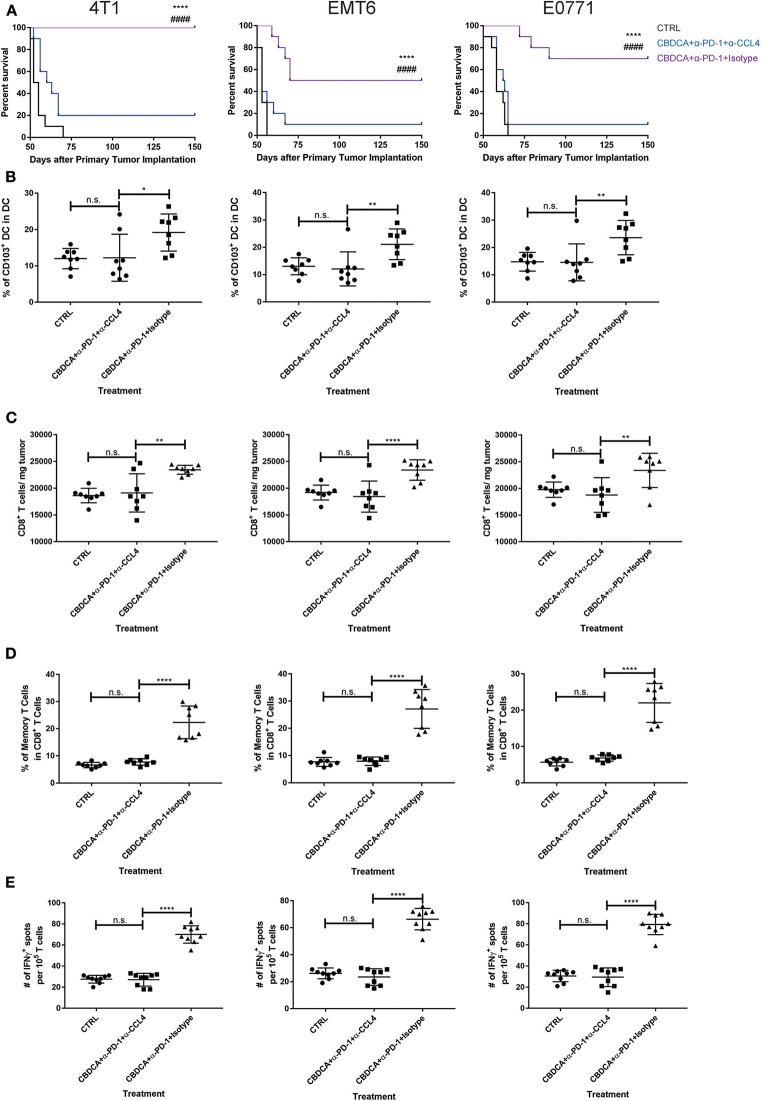
The anti-cancer effect of CBDCA and anti-PD-1 antibodies in the secondary tumor is CCL4-dependent. Tumor-bearing mice treated with CBDCA and anti-PD-1 were administered with anti-CCL4 antibodies or respective isotype control antibodies as described in Methods. Left columns: 4T1; middle columns: EMT6; right columns: E0771. **(A)** Administration of anti-CCL4 antibodies impaired the survival rates of mice receiving CBDCA combined with anti-PD-1. The symbol “*” denotes the difference between CBDCA+α-PD-1+Isotype and CTRL. The symbol “#” denotes the difference between CBDCA+α-PD-1+Isotype and CBDCA+α-PD-1+α-CCL4. ^****, *####*^*p* < 0.0001 by log-rank tests, *n* = 10 per group from one of triplicated experiments. **(B)** CCL4 neutralization reduced the abundance of CD103^+^ DC seen in mice treated with CBDCA and anti-PD-1 antibodies. **(C)** Administration of CCL4 neutralizing antibodies decreased the proportion of CD8^+^ T cells in the tumor. **(D)** CCL4 neutralization decreased the proportion of memory CD8^+^ T cells compared to mice treated with CBDCA and anti-PD-1 antibodies. **(E)** Blocking CCL4 inhibits the elevation of tumor-specific CD8^+^ T cells in mice treated with CBDCA and anti-PD-1 antibodies measured by ELISpot assay. **(B–E)** *n* = 8 per group from one of triplicated experiments, compared by one-way ANOVA with Bonferroni *post-hoc* tests. n.s., no significance; *p < 0.05; **p < 0.01; ****p < 0.0001.

Finally, we asked whether blocking the CCL4 signaling could abolish the recruitment of CD103^+^ DC and CD8^+^ T cells in the tumor, and subsequent anti-cancer effects. Anti-CCL4 neutralizing antibodies were applied as described in Methods. As shown in Figure 8A, indeed, administration of anti-CCL4 neutralizing antibodies impaired the survival rates of mice treated with CBDCA and anti-PD-1 antibodies before surgery. We then assessed the abundance of CD103^+^ DC and CD8^+^ T cells in the tumor microenvironment. As illustrated in Figures 8B,C, administration of anti-CCL4 antibodies reduced the abundance of CD103^+^ DC and CD8^+^ T cells that have been observed in mice with immunochemotherapy before surgery in all three models. Moreover, blocking CCL4 inhibited the increase of memory CD8^+^ T cells (Figure 8D) and tumor-specific CD8^+^ T cells (Figure 8E) mediated by the combination of CBDCA and anti-PD-1 antibodies in the 4T1 tumors, as well as other two models. These data indicate that immunochemotherapy of CBDCA combined with anti-PD-1 antibodies increases the expression of CCL4 in the secondary tumor, which subsequently to recruit CD103^+^ DC and CD8^+^ T cells to mediate the tumor-killing effect in TNBC models.

## Discussion

In the present study, we demonstrate that the combination of CBDCA and anti-PD-1 antibodies as an immunochemotherapy option effectively eliminates secondary murine TNBC after surgery. More importantly, immunochemotherapy of CBDCA and anti-PD-1 antibodies before surgery indicates a sustainable influence for controlling a secondary tumor. This sustainable protection after surgery is CD8^+^ T cell-dependent, which requires CCL4 expressed in the tumor and involves CD103^+^ DC recruited by CCL4. This study highlights the potential application of neoadjuvant therapy of CBDCA and anti-PD-1 in TNBC patients, and the critical roles of CCL4 and CD103^+^ DC in CD8^+^ T cell priming and infiltration in the tumor microenvironment.

TNBC as a special type of breast cancer has gained a great deal of attention from oncologists and basic researchers. Patients with TNBC have an unfavorable prognosis, even with radical surgery and chemotherapy, especially during the first 3 years after diagnosis (29). The major cause of death is recurrence and metastasis. Neoadjuvant therapy provides an option for breast cancer patients with advanced stages and/or aggressive histology types, but the 5-year survival rate is still suboptimal. Especially, the “TNBC paradox,” where TNBC patients have an increased pathologic complete response rate whereas the overall survival has not been prolonged (30), highlights new therapeutic options like immunotherapy are needed for TNBC patients. In this study, we present further evidence supporting the idea of combining cytotoxic agents and anti-cancer immunotherapy as an approach for pre-surgery treatment. Not surprisingly, treatment with CBDCA and anti-PD-1 induced tumor shrinking at the early time points (Figure 2), but could only improve the survival rates by a limited degree. This phenomenon is in line with clinical observation for patients with TNBC (31, 32) and mouse models (18), which indicates the importance to solve this predicament.

In order to improve the prognosis of TNBC, we tested the idea of using the combination of CBDCA and anti-PD-1 antibodies as immunochemotherapy before surgery. As shown in Figure 3, this therapy using CBDCA and anti-PD-1 effectively kills the secondary tumor, and maintains sustainable protection, which has been demonstrated as remarkably improved long-term survival rates. In one of the three TNBC models used in the present study—the 4T1 model, the cure rate for the secondary tumor was 90% (defined as tumor-free for longer than 150 days after the primary tumor implantation, Figure 3B), which is an encouraging observation compared to the only 10% cure rate in mice bearing the primary 4T1 tumors. Similar protection occurred through all three pre-clinical models. These observations have raised the possibilities to use immunochemotherapy with CBDCA and anti-PD-1 antibodies in clinical practice for TNBC patients.

**Figure 9:**
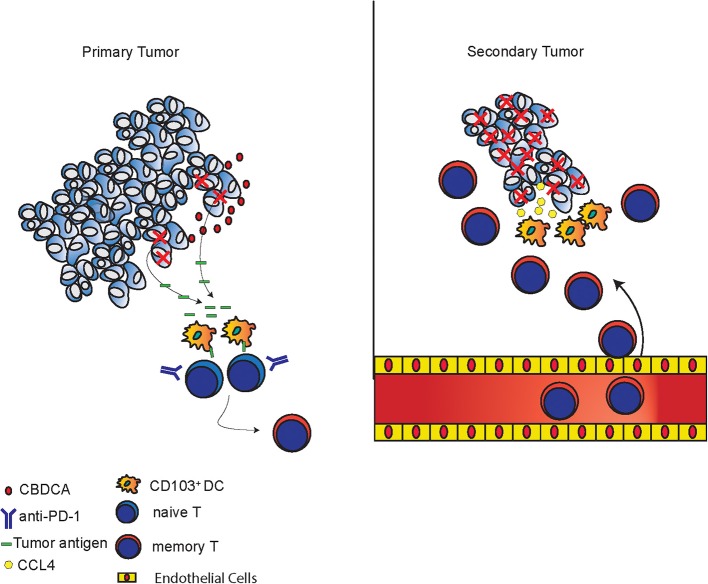
Proposed mechanisms of anti-cancer effects induced by the combination of CBDCA and anti-PD-1 antibodies. In primary tumors, CBDCA causes tumor cell death, which releases tumor antigens. CD103^+^ DC uptake tumor antigens and present them to naive T cells to produce memory T cells, which have enhanced function as a result of PD-1 blockade. In secondary tumors, CCL4 secreted by TNBC cells recruits CD103^+^ DC, which subsequently involve tumor-specific CD8^+^ T cells to the tumor microenvironment to kill cancer cells.

The mechanisms of tumor killing mediated by CBDCA and anti-PD-1 in the setting of neoadjuvant therapy are complex, which have been explored in the present study. During the treatment of the primary tumor, CBDCA induces tumor cell death (Figure 1 and Supplemental Figure 2) and anti-PD-1 antibodies induce a very limited degree of necrosis (Supplemental Figure 2), which probably may release tumor-specific antigens to the tumor microenvironment. CD103^+^ DC uptake and process tumor antigens, and subsequently present tumor antigens toward activated T cells to produce tumor-specific CD8^+^ T cells. Administration of anti-PD-1 antibodies unleashes T cells functions by inhibiting immune checkpoints. Last, during the stage of the secondary tumor, the existing effect of neoadjuvant therapy increases CCL4 expressed in the secondary tumor with an unexplored mechanism, which is responsible for recruiting CD103^+^ DC. CD103^+^ DC subsequently mediate tumor-specific CD8^+^ T cells infiltrating to the tumor microenvironment to execute anti-cancer effects. The proposed mechanism is presented in Figure 9.

One question that has arisen from the present project is why the regimen is very effective in the secondary tumor, but less efficient in the primary tumor. It has been reported that the efficacy of anti-tumor immunotherapy mediated by immune checkpoint inhibitors depends on the tumor burden (33). After the treatment during the primary tumor period, the host has undergone the process of producing tumor-specific T cells. At the time of the secondary tumor, memory T cells specific to tumor antigens are circulating in the system. Meanwhile, the size of secondary tumors is relatively small, therefore it is reasonable to speculate that memory T cells are capable to overcome the relatively smaller tumor burden to maintain a tumor-free status.

We document the importance of CD8^+^ T cells and CD103^+^ DC during neoadjuvant immunotherapy with CBDCA and anti-PD1 antibodies in TNBC-bearing mice. However, it is still possible that other immune cell types are involved in this protection given the complexity of the tumor microenvironment. For instance, it has been reported (34) that macrophages in the TNBC have a supportive role for tumor growth by the following mechanisms: (1) production of anti-inflammatory cytokines and other factors; (2) inhibition of T cell functions in the tumor microenvironment; (3) induction of regulatory T cell differentiation; and (4) upregulation of immune checkpoints like PD-1. Therefore, it is worth exploring the roles of macrophages in the setting of neoadjuvant immunotherapy for TNBC. T help (Th) 1 cells and regulatory T cells (Treg) are also important players in the tumor microenvironment during anti-cancer immunotherapy. Accumulation of CXCR3^+^ Th1 cells into the tumor microenvironment delays implanted tumors in mice treated with anti-PD-1 antibodies (35). FoxP3^+^ Treg are an immunoregulatory Th cell type that downgrades immune responses. PD-1 is highly expressed on the surface of Treg and has a key role in controlling the functions of Treg (36). Treg is a target cell type of anti-PD1 antibody therapy, as administration of this antibody inhibits Treg induction and proliferation to enhance anti-tumor killing (37). However, the roles of Th1 and/or Treg in the context of neoadjuvant immunochemotherapy require further investigations.

The present study has a few potential pitfalls. First, we did not explore the mechanism where expression of CCL4 by tumor cells is increased. We speculate that memory T cells recruited to the tumor microenvironment by sensing the tumor antigens trigger a feedforward loop, which is composed of increased CCL4, recruited CD103^+^DC, and T cell infiltration. This cycle requires every element. However, the precise mechanism still remains elusive. Second, we did not test whether administration of anti-PD-1 antibodies and/or CBDCA after surgery as a supplement to neoadjuvant immuochemotherapy has any benefit for survival. It is possible that adjuvant therapy of anti-PD-1 antibodies and/or CBDCA might have some additional survival advantages, but the side-effects and cytotoxicity observed in patients treated with immune checkpoint inhibitors (38) may limit the application of this approach. Third, we used an implanted relapse cancer model, rather than a spontaneous recurrence model. According to our preliminary results for mice undergoing neoadjuvant immunotherapy and surgery (without implantation of the secondary tumor), the recurrence rate was as low as 10% (data not shown). This low recurrence prevalence does not reflect clinical observations. This pitfall of the present study also calls for new murine TNBC models. Last, we have not determined the efficacy of neoadjuvant therapy with CBDCA and anti-PD-1 antibodies in TNBC patients. However, this study has definitely shed insight into the probable tumor-killing effects in clinical settings, which will be tested in the near future.

Immunochemotherapy with CBDCA and anti-PD-1 antibodies before surgery present sustainable protection against secondary tumors by increasing CCL4 expression in the tumor and subsequently CD103^+^DC and CD8^+^ T cells infiltration in the tumor microenvironment in mouse TNBC models.

## Data Availability Statement

All datasets generated for this study are included in the article/Supplementary Material.

## Ethics Statement

This study was carried out in accordance with the recommendations of Declaration of Helsinki. The protocol was approved by the Ethics Committee of Fourth Affiliated Hospital of Harbin Medical University.

## Author Contributions

MG was responsible for conceptualization, supervision of the experimental process, and review the draft. TW was responsible for funding application and preparation of the first draft of the manuscript. LJ was responsible for most experiments and data collection. SB was responsible for cell culture. LT was responsible for data analysis and illustration. HS was consulted as a senior researcher. All authors have read the final version of this manuscript and agreed to publish the current study.

### Conflict of Interest

The authors declare that the research was conducted in the absence of any commercial or financial relationships that could be construed as a potential conflict of interest.

## Supplementary Material

Supplemental Figure 1 Administration of CBDCA increases the levels of necrosis in PDX from additional three patients. PDX collected from additional three TNBC patients were planted to NOD/SCID mice, and CBDCA was administered as planned. Seventy-two hours after treatment, CBDCA increased the levels of necrosis of PDX collected from Patient 201821212 [52 years old, *n* = 9 mice **(A)** 15 days to reach the designated volume], Patient 201821344 [65 years old, *n* = 8 mice **(B)** 29 days to reach the designated volume], and Patient 201711434 [53 years old, *n* = 9 mice **(C)** 32 days to reach the designated volume]. **(D)** Pooled data of four patients involved in this project indicated increased numbers of necrotic tumor cells. ^****^*p* < 0.0001, by student *t*-tests.

Supplemental Figure 2 Treatment with CBDCA and anti-PD-1 antibodies increases levels of necroptosis in the tumor. Necroptotic cells in the implanted cancer were determined by RIP3 staining by immunofluorescence at day 24 after the initial implantation. **(A)** Representative images of RIP3 staining. scale bar: 43 μm. Treatment of CBDCA and α-PD-1 antibodies increased levels of necroptosis in 4T1 **(B)**, EMT6 **(C)**, and E0771 **(D)** tumors. *n* = 10 per group. ^****^*p* < 0.0001, by one-way ANOVA with Bonferroni *post-hoc* tests.

Supplemental Figure 3 Tumor metastasis patterns of implanted TNBC models after treatment. **(A)** 4T1 tumors. **(B)** EMT6 tumors. **(C)** E0771 tumors. *n* = 10 in each group from one of triplicated experiments. No significance was observed by Chi-square tests.

Supplemental Figure 4 Correlations between the abundance of memory CD8^+^ T cells (% of Tm) and tumor volume. The abundance of memory T cells was negatively associated with the tumor volume. **(A)** 4T1 tumors. **(B)** EMT6 tumors. **(C)** E0771 tumors. Figures are representative results from one of triplicated experiments, which were analyzed by the linear regression.

Supplemental Figure 5 The percentage of CD103^+^ DC in CD45^+^ cells in the tumor microenvironment is increased in those treated with CBDCA and α-PD-1. *n* = 8 per group from one of triplicated experiments. ^**^*p* < 0.01; ^***^*p* < 0.001, by two-tail student *t*-tests.

Supplemental Figure 6 Correlations between the abundance of CD103^+^ DC cells and tumor volumes. The abundance of CD103^+^ DC was negatively associated with the tumor volume of the secondary tumor at Day 35 after the primary tumor implantation. **(A)** 4T1 tumors. **(B)** EMT6 tumors. **(C)** E0771 tumors. Figures are representative results from one of triplicated experiments, which were analyzed by the linear regression.

Supplemental Figure 7 Correlations between the abundance of CCL4^+^ tumor cells and tumor volumes. The abundance of CCL4^+^ tumor cells was negatively associated with the tumor volume of the secondary tumor at Day 35 after the primary tumor implantation. **(A)** 4T1 tumors. **(B)** EMT6 tumors. **(C)** E0771 tumors. Figures are representative results from one of triplicated experiments, which were analyzed by the linear regression.

Supplemental Figure 8 Treatment with CBDCA and anti-PD-1 antibodies has no effects in the abundance of CD45^+^CCL4^+^ cells in the secondary tumor at Day 35. **(A)** 4T1 tumors. **(B)** EMT6 tumors. **(C)** E0771 tumors. n.s., no significance, by two-tailed student *t*-tests.

Supplemental Figure 9 Correlations between the abundance of CD45^+^CCL4^+^ cells and tumor volumes. The abundance of CD45^+^CCL4^+^ cells was not associated with the tumor volume of the secondary tumor at Day 35 after the primary tumor implantation. **(A)** 4T1 tumors. **(B)** EMT6 tumors. **(C)** E0771 tumors. Figures are representative results from one of triplicated experiments, which were analyzed by the linear regression.

Supplemental Table 1 Volume of primary 4T1 tumors at Day 24 after implantation.

Supplemental Table 2 Volume of primary EMT6 tumors at Day 24 after implantation.

Supplemental Table 3 Volume of primary E0771 tumors at Day 24 after implantation.

Supplemental Table 4 Survival of primary 4T1 tumor-bearing mice.

Supplemental Table 5 Survival of primary EMT6 tumor-bearing mice.

Supplemental Table 6 Survival of primary E0771 tumor-bearing mice.

Supplemental Table 7 Volume of secondary 4T1 tumors at Day 48 after primary implantation.

Supplemental Table 8 Volume of secondary EMT6 tumors at Day 48 after primary implantation.

Supplemental Table 9 Volume of secondary E0771 tumors at Day 48 after primary implantation.

Supplemental Table 10 Survival of secondary 4T1 tumor-bearing mice.

Supplemental Table 11 Survival of secondary EMT6 tumor-bearing mice.

Supplemental Table 12 Survival of secondary E0771 tumor-bearing mice.
