# Supplementary figures and images for: Therapy With Carboplatin and Anti-PD-1 Antibodies Before Surgery Demonstrates Sustainable Anti-Tumor Effects for Secondary Cancers in Mice With Triple-Negative Breast Cancer
Source: Front Immunol. 2020 Mar 5;11:366. doi: 10.3389/fimmu.2020.00366 (PMC7066228; doi:10.3389/fimmu.2020.00366)

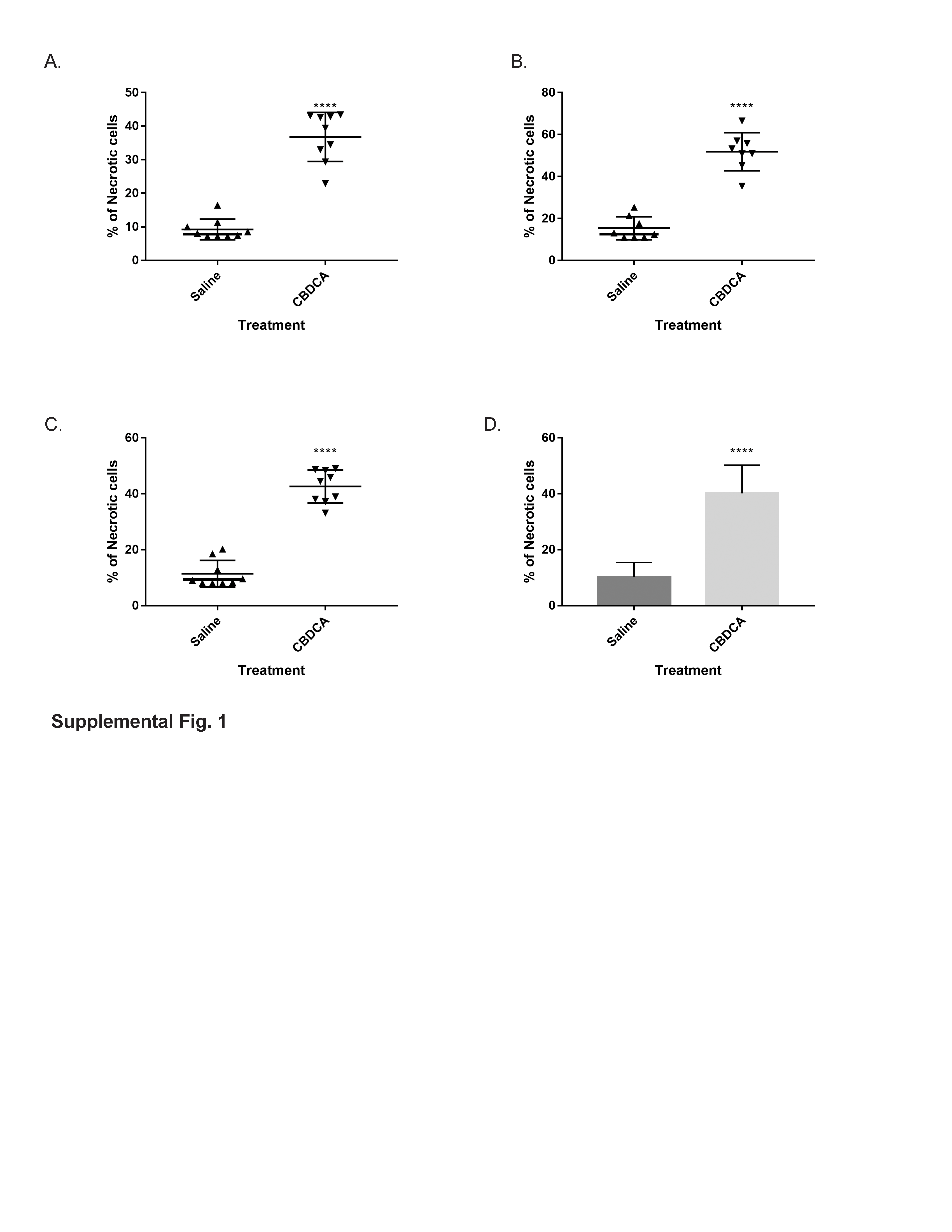

Supplement: Supplemental Figure 1 — Administration of CBDCA increases the levels of necrosis in PDX from additional three patients. PDX collected from additional three TNBC patients were planted to NOD/SCID mice, and CBDCA was administered as planned. Seventy-two hours after treatment, CBDCA increased the levels of necrosis of PDX collected from Patient 201821212 [52 years old, n = 9 mice (A) 15 days to reach the designated volume], Patient 201821344 [65 years old, n = 8 mice (B) 29 days to reach the designated volume], and Patient 201711434 [53 years old, n = 9 mice (C) 32 days to reach the designated volume]. (D) Pooled data of four patients involved in this project indicated increased numbers of necrotic tumor cells. ****p < 0.0001, by student t-tests. [file Image_1.TIFF]

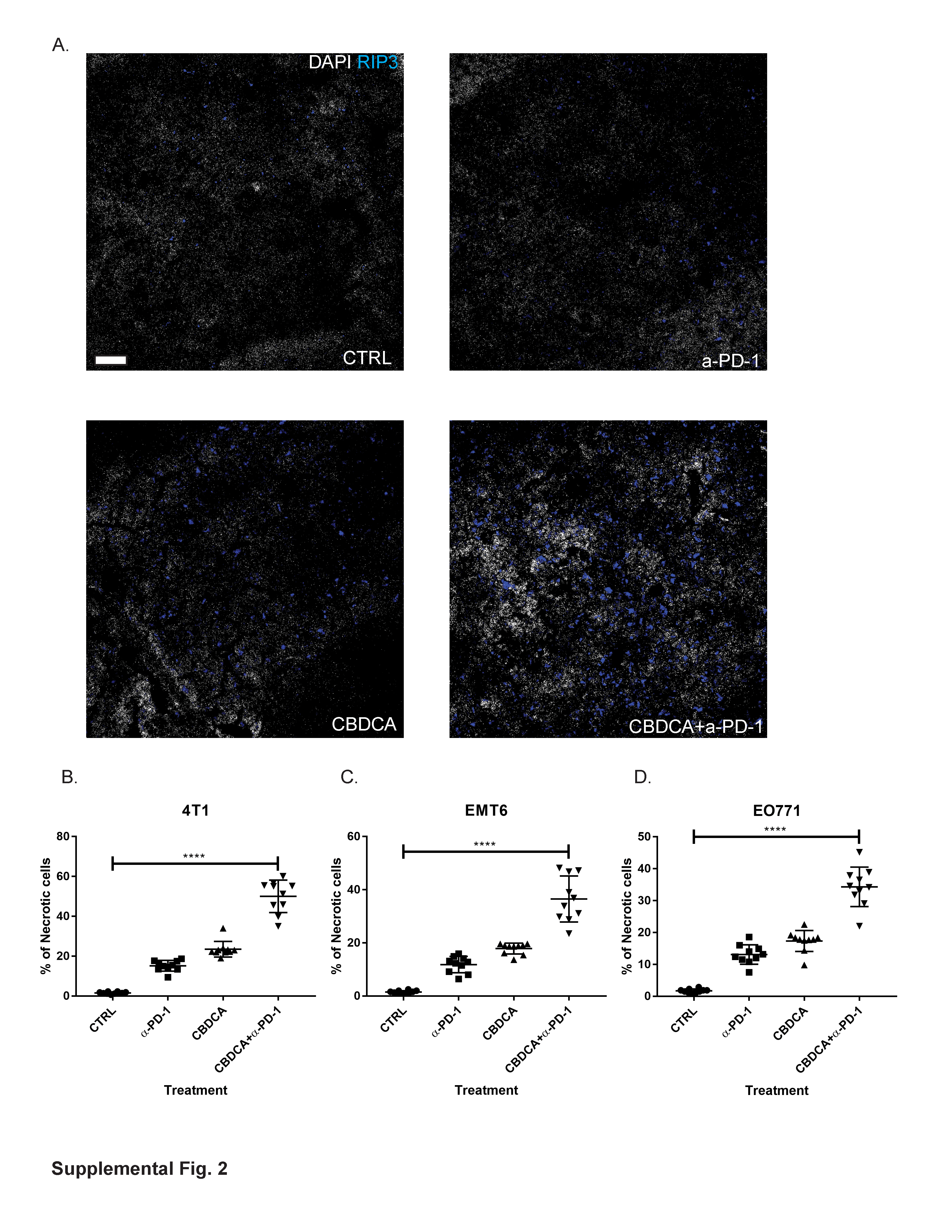

Supplement: Supplemental Figure 2 — Treatment with CBDCA and anti-PD-1 antibodies increases levels of necroptosis in the tumor. Necroptotic cells in the implanted cancer were determined by RIP3 staining by immunofluorescence at day 24 after the initial implantation. (A) Representative images of RIP3 staining. scale bar: 43 μm. Treatment of CBDCA and α-PD-1 antibodies increased levels of necroptosis in 4T1 (B), EMT6 (C), and E0771 (D) tumors. n = 10 per group. ****p < 0.0001, by one-way ANOVA with Bonferroni post-hoc tests. [file Image_2.TIFF]

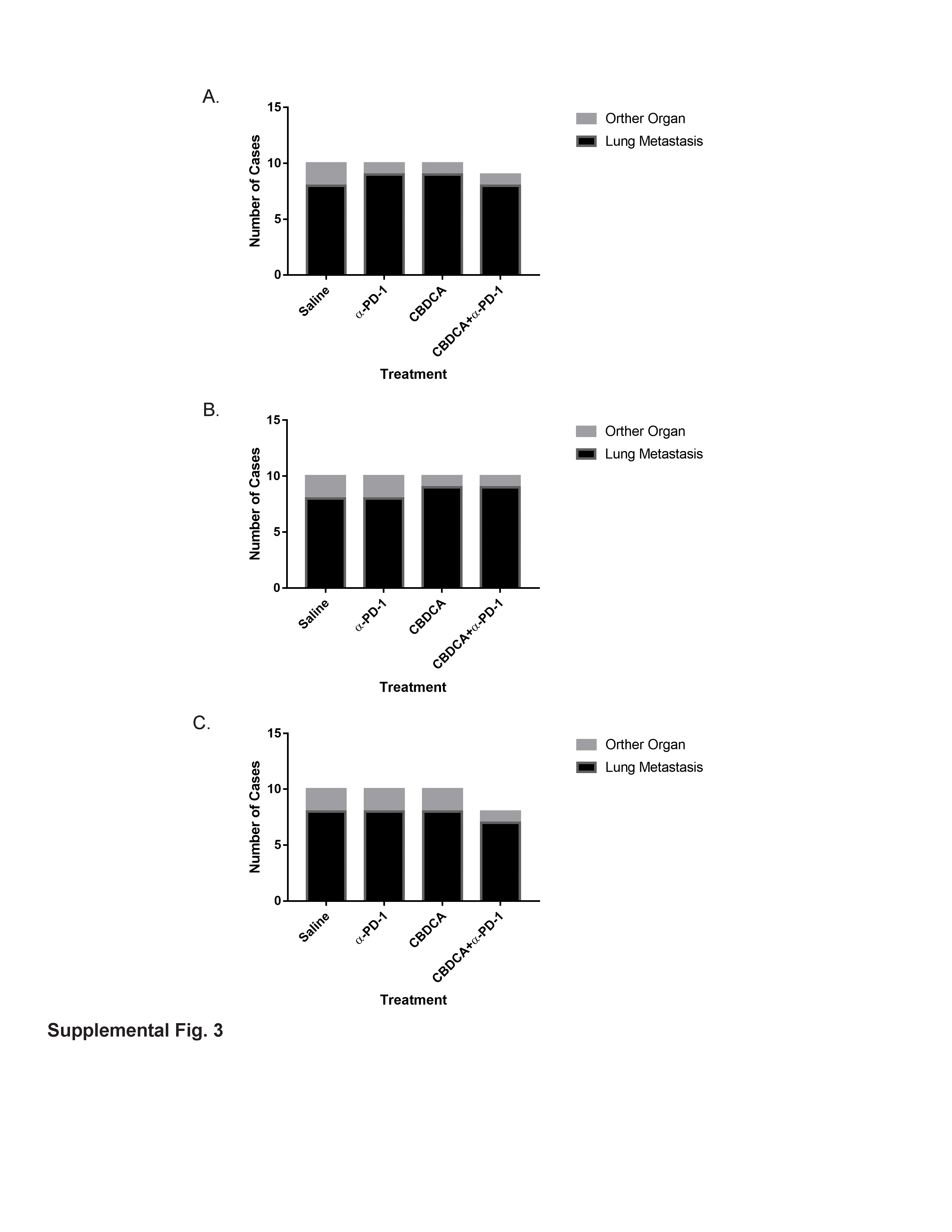

Supplement: Supplemental Figure 3 — Tumor metastasis patterns of implanted TNBC models after treatment. (A) 4T1 tumors. (B) EMT6 tumors. (C) E0771 tumors. n = 10 in each group from one of triplicated experiments. No significance was observed by Chi-square tests. [file Image_3.TIFF]

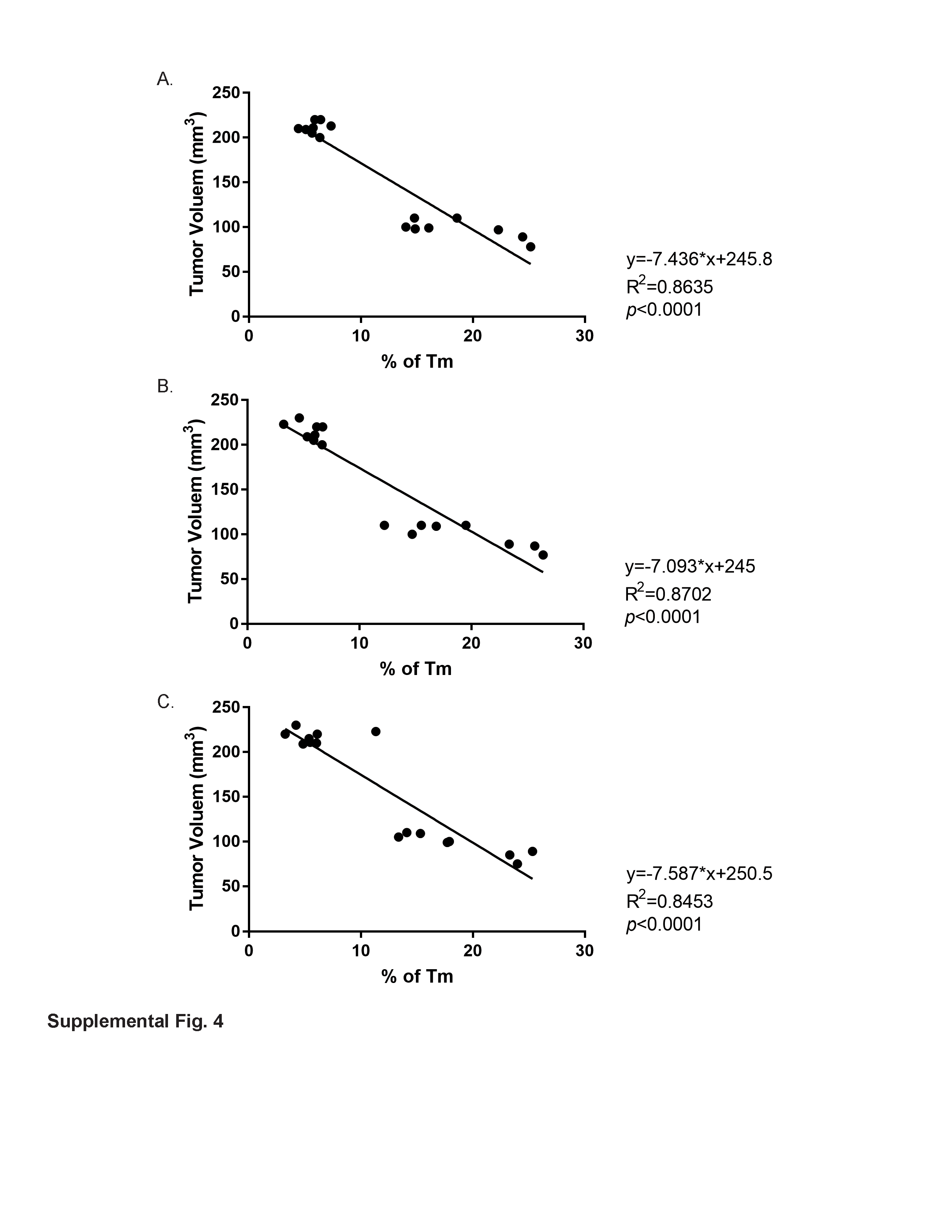

Supplement: Supplemental Figure 4 — Correlations between the abundance of memory CD8+ T cells (% of Tm) and tumor volume. The abundance of memory T cells was negatively associated with the tumor volume. (A) 4T1 tumors. (B) EMT6 tumors. (C) E0771 tumors. Figures are representative results from one of triplicated experiments, which were analyzed by the linear regression. [file Image_4.TIFF]

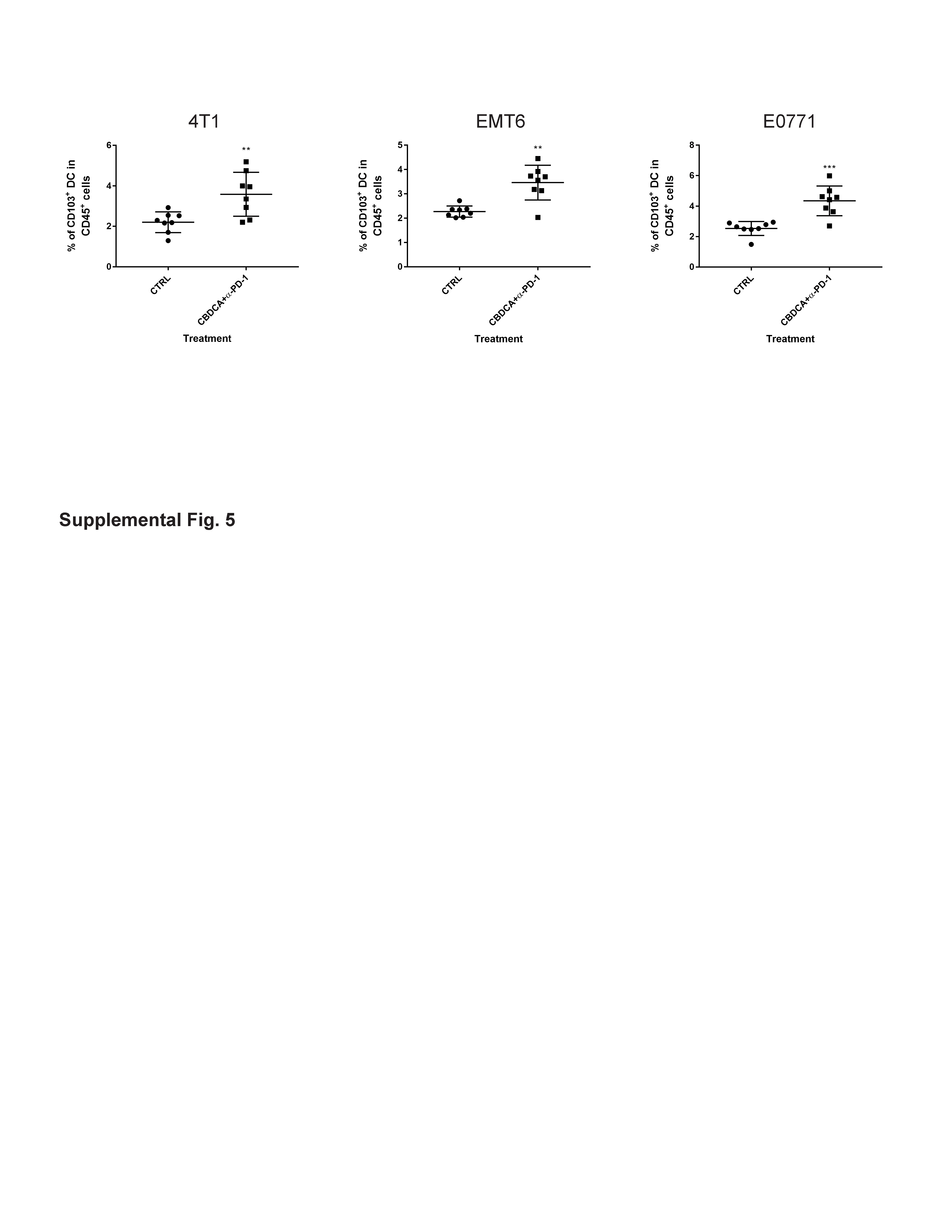

Supplement: Supplemental Figure 5 — The percentage of CD103+ DC in CD45+ cells in the tumor microenvironment is increased in those treated with CBDCA and α-PD-1. n = 8 per group from one of triplicated experiments. **p < 0.01; ***p < 0.001, by two-tail student t-tests. [file Image_5.TIFF]

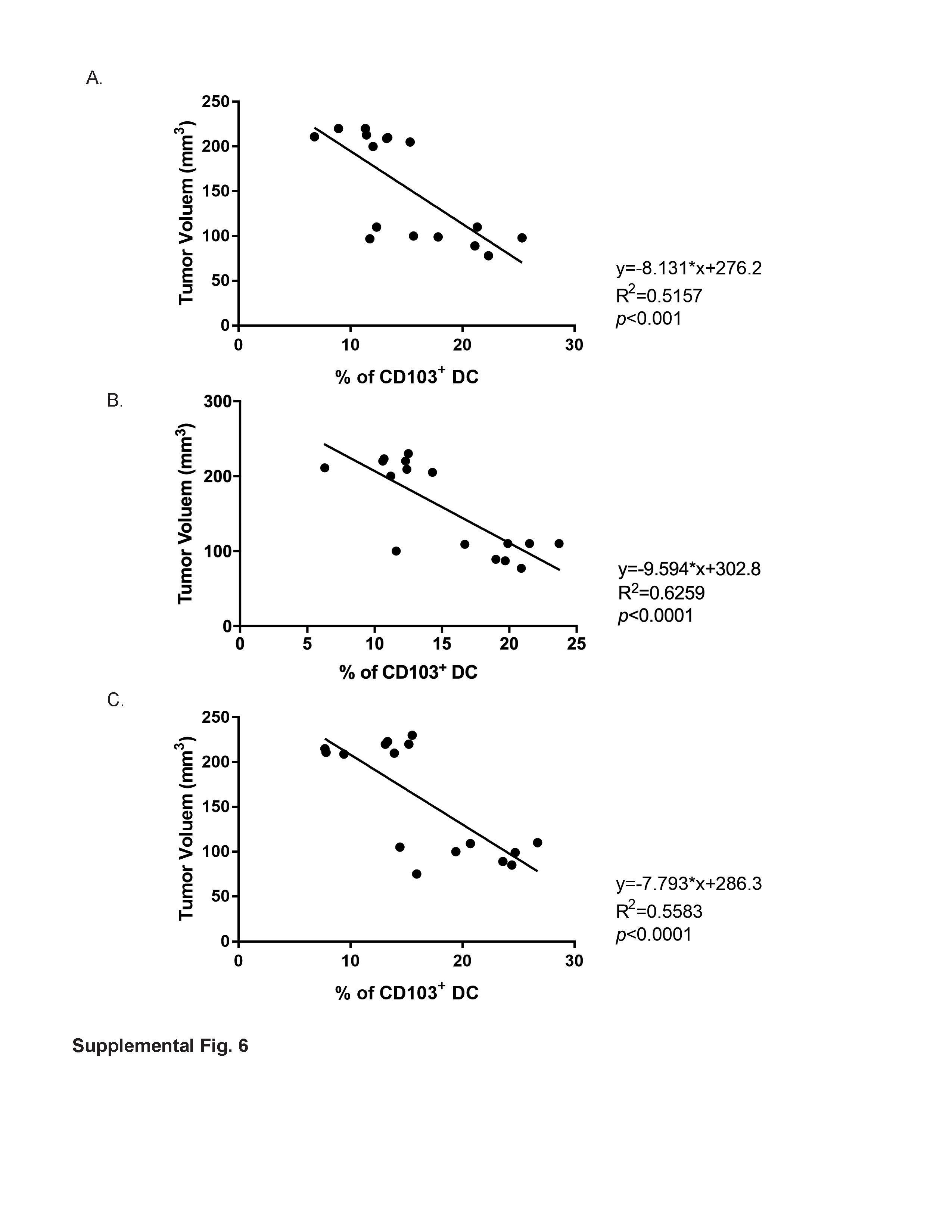

Supplement: Supplemental Figure 6 — Correlations between the abundance of CD103+ DC cells and tumor volumes. The abundance of CD103+ DC was negatively associated with the tumor volume of the secondary tumor at Day 35 after the primary tumor implantation. (A) 4T1 tumors. (B) EMT6 tumors. (C) E0771 tumors. Figures are representative results from one of triplicated experiments, which were analyzed by the linear regression. [file Image_6.TIFF]

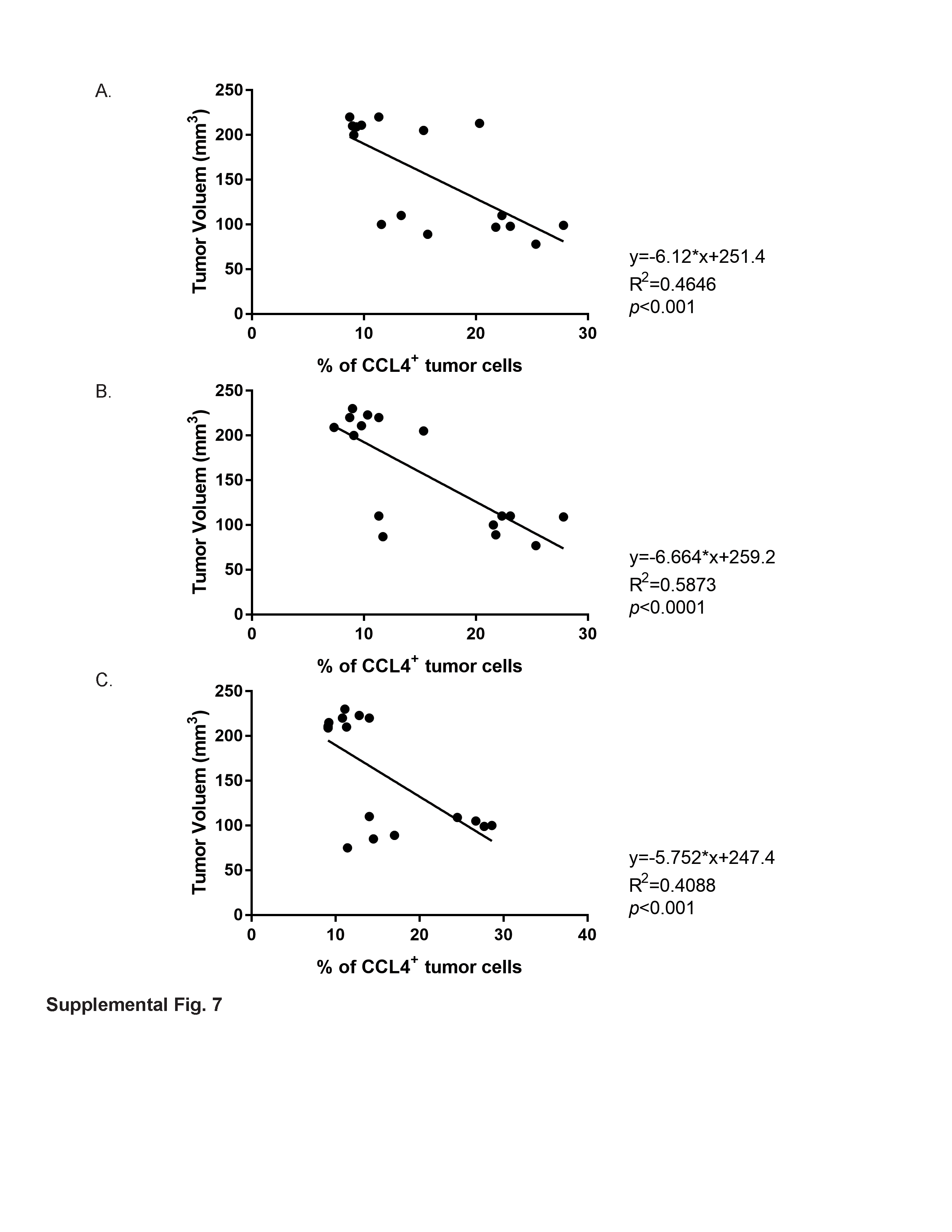

Supplement: Supplemental Figure 7 — Correlations between the abundance of CCL4+ tumor cells and tumor volumes. The abundance of CCL4+ tumor cells was negatively associated with the tumor volume of the secondary tumor at Day 35 after the primary tumor implantation. (A) 4T1 tumors. (B) EMT6 tumors. (C) E0771 tumors. Figures are representative results from one of triplicated experiments, which were analyzed by the linear regression. [file Image_7.TIFF]

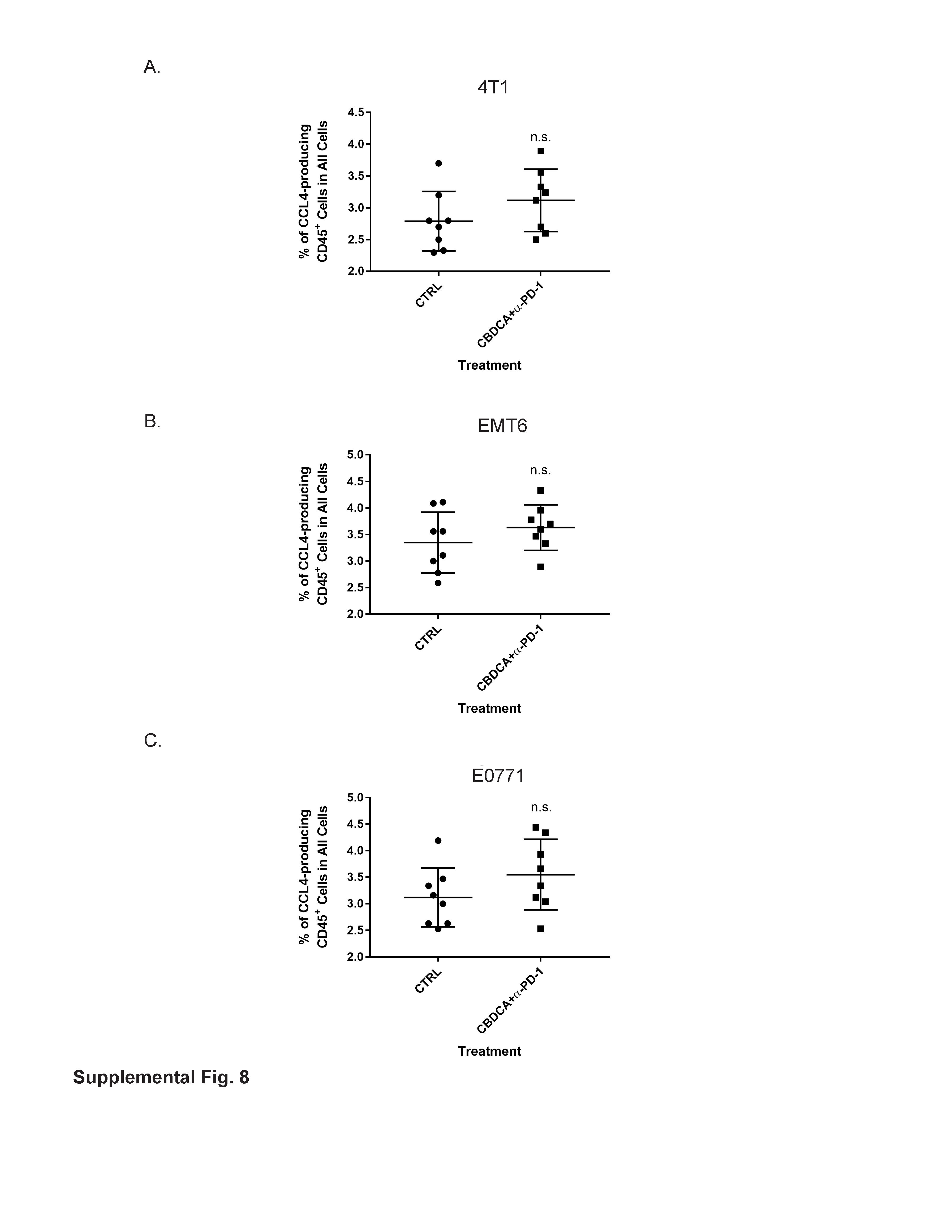

Supplement: Supplemental Figure 8 — Treatment with CBDCA and anti-PD-1 antibodies has no effects in the abundance of CD45+CCL4+ cells in the secondary tumor at Day 35. (A) 4T1 tumors. (B) EMT6 tumors. (C) E0771 tumors. n.s., no significance, by two-tailed student t-tests. [file Image_8.TIFF]

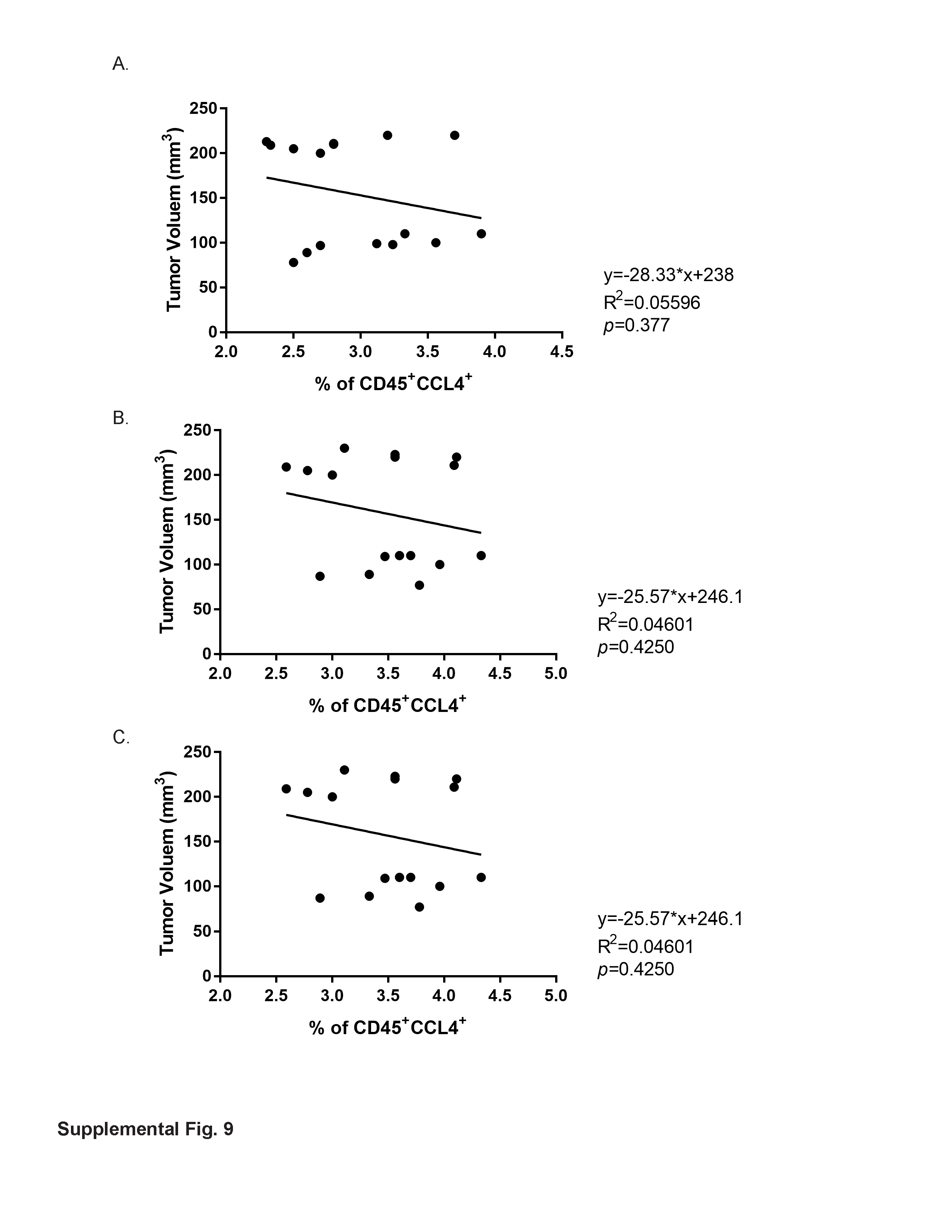

Supplement: Supplemental Figure 9 — Correlations between the abundance of CD45+CCL4+ cells and tumor volumes. The abundance of CD45+CCL4+ cells was not associated with the tumor volume of the secondary tumor at Day 35 after the primary tumor implantation. (A) 4T1 tumors. (B) EMT6 tumors. (C) E0771 tumors. Figures are representative results from one of triplicated experiments, which were analyzed by the linear regression. [file Image_9.TIFF]
