# Supplementary material for: Therapy With Carboplatin and Anti-PD-1 Antibodies Before Surgery Demonstrates Sustainable Anti-Tumor Effects for Secondary Cancers in Mice With Triple-Negative Breast Cancer
Source: Front Immunol. 2020 Mar 5;11:366. doi: 10.3389/fimmu.2020.00366 (PMC7066228; doi:10.3389/fimmu.2020.00366)
Supplement: Supplemental Table 1 — Volume of primary 4T1 tumors at Day 24 after implantation. [file Table_1.DOCX]

Supplemental Table 1: Volume of primary 4T1 tumors at Day 24 after implantation

|  | Tumor volume  (mm^3^, mean±SD) | *P* values | |
| --- | --- | --- | --- |
|  |  | v.s. CTRL | v.s. CBDCA+α-PD-1 |
| CTRL | 1268±85.74 |  | <0.0001 |
| α-PD-1 | 784±119 | <0.05 | <0.0001 |
| CBDCA | 922.9±78.84 | <0.05 | <0.0001 |
| CBDCA+α-PD-1 | 508±78.15 | <0.0001 |  |

Note: Statistical analyses were performed using one-way ANOVA with Bonferroni post hoc tests. The same experiments as Figure 2.

Supplemental Table 2: Volume of primary EMT6 tumors at Day 24 after implantation

|  | Tumor volume  (mm^3^, mean±SD) | *P* values | |
| --- | --- | --- | --- |
|  |  | v.s. CTRL | v.s. CBDCA+α-PD-1 |
| CTRL | 1285±335.2 |  | <0.0001 |
| α-PD-1 | 925±78.75 | <0.05 | <0.0001 |
| CBDCA | 963±232.5 | <0.05 | <0.0001 |
| CBDCA+α-PD-1 | 570±109 | <0.0001 |  |

Note: Statistical analyses were performed using one-way ANOVA with Bonferroni post hoc tests. The same experiments as Figure 2.

Supplemental Table 3: Volume of primary E0771 tumors at Day 24 after implantation

|  | Tumor volume  (mm^3^, mean±SD) | *P* values | |
| --- | --- | --- | --- |
|  |  | v.s. CTRL | v.s. CBDCA+α-PD-1 |
| CTRL | 999±260.6 |  | <0.0001 |
| α-PD-1 | 718±61.08 | <0.05 | <0.0001 |
| CBDCA | 748±180.9 | <0.05 | <0.0001 |
| CBDCA+α-PD-1 | 459±88.75 | <0.0001 |  |

Note: Statistical analyses were performed using one-way ANOVA with Bonferroni post hoc tests. The same experiments as Figure 2.

Supplemental Table 4: Survival of primary 4T1 tumor-bearing mice.

|  | Median Survival (d) | Survival Rate (%) | *P* values | |
| --- | --- | --- | --- | --- |
|  |  |  | v.s. CTRL | v.s. CBDCA+α-PD-1 |
| CTRL | 31 | 0 |  | <0.0001 |
| α-PD-1 | 40 | 0 | n.s. | <0.0001 |
| CBDCA | 35 | 0 | n.s. | <0.0001 |
| CBDCA+α-PD-1 | 45 | 10 | <0.0001 |  |

Note: Survivals were analyzed by the Kaplan-Meier method and compared by the log-rank test. n.s., no significance. The same experiments as Figure 2.

Supplemental Table 5: Survival of primary EMT6 tumor-bearing mice.

|  | Median Survival (d) | Survival Rate (%) | *P* values | |
| --- | --- | --- | --- | --- |
|  |  |  | v.s. CTRL | v.s. CBDCA+α-PD-1 |
| CTRL | 28 | 0 |  | <0.001 |
| α-PD-1 | 35 | 0 | n.s. | <0.001 |
| CBDCA | 33 | 0 | n.s. | <0.001 |
| CBDCA+α-PD-1 | 40 | 0 | <0.001 |  |

Note: Survivals were analyzed by the Kaplan-Meier method and compared by the log-rank test. n.s., no significance. The same experiments as Figure 2.

Supplemental Table 6: Survival of primary E0771 tumor-bearing mice.

|  | Median Survival (d) | Survival Rate (%) | *P* values | |
| --- | --- | --- | --- | --- |
|  |  |  | v.s. CTRL | v.s. CBDCA+α-PD-1 |
| CTRL | 31 | 0 |  | <0.0001 |
| α-PD-1 | 42 | 0 | n.s. | <0.0001 |
| CBDCA | 35 | 0 | n.s. | <0.0001 |
| CBDCA+α-PD-1 | 52 | 20 | <0.0001 |  |

Note: Survivals were analyzed by the Kaplan-Meier method and compared by the log-rank test. n.s., no significance. The same experiments as Figure 2.

Supplemental Table 7: Volume of secondary 4T1 tumors at Day 48 after primary implantation

|  | Tumor volume  (mm^3^, mean±SD) | *P* values | |
| --- | --- | --- | --- |
|  |  | v.s. CTRL | v.s. CBDCA+α-PD-1 |
| CTRL | 1182±86.13 |  | <0.0001 |
| α-PD-1 | 718±109.1 | <0.05 | <0.0001 |
| CBDCA | 904.9±158.9 | <0.05 | <0.0001 |
| CBDCA+α-PD-1 | 465.3±71.77 | <0.0001 |  |

Note: Statistical analyses were performed using one-way ANOVA with Bonferroni post hoc tests. The same experiments as Figure 3.

Supplemental Table 8: Volume of secondary EMT6 tumors at Day 48 after primary implantation

|  | Tumor volume  (mm^3^, mean±SD) | *P* values | |
| --- | --- | --- | --- |
|  |  | v.s. CTRL | v.s. CBDCA+α-PD-1 |
| CTRL | 1223±326.8 |  | <0.0001 |
| α-PD-1 | 830.8±99.43 | <0.05 | <0.0001 |
| CBDCA | 993.2±199.5 | n.s. | <0.0001 |
| CBDCA+α-PD-1 | 533.6±102 | <0.0001 |  |

Note: Statistical analyses were performed using one-way ANOVA with Bonferroni post hoc tests. n.s., no significance. The same experiments as Figure 3.

Supplemental Table 9: Volume of secondary E0771 tumors at Day 48 after primary implantation

|  | Tumor volume  (mm^3^, mean±SD) | *P* values | |
| --- | --- | --- | --- |
|  |  | v.s. CTRL | v.s. CBDCA+α-PD-1 |
| CTRL | 905.6±235.3 |  | <0.0001 |
| α-PD-1 | 561.9±217.4 | <0.05 | <0.0001 |
| CBDCA | 841.7±287.3 | n.s. | <0.0001 |
| CBDCA+α-PD-1 | 412.2±80.56 | <0.0001 |  |

Note: Statistical analyses were performed using one-way ANOVA with Bonferroni post hoc tests. n.s., no significance. The same experiments as Figure 3.

Supplemental Table 10: Survival of secondary 4T1 tumor-bearing mice.

|  | Median Survival (d) | Survival Rate (%) | *P* values | |
| --- | --- | --- | --- | --- |
|  |  |  | v.s. CTRL | v.s. CBDCA+α-PD-1 |
| CTRL | 55 | 0 |  | <0.0001 |
| α-PD-1 | 65 | 0 | n.s. | <0.0001 |
| CBDCA | 58 | 0 | n.s. | <0.0001 |
| CBDCA+α-PD-1 | Not Reached | 90 | <0.0001 |  |

Note: Survivals were analyzed by the Kaplan-Meier method and compared by the log-rank test. n.s., no significance. The same experiments as Figure 3.

Supplemental Table 11: Survival of secondary EMT6 tumor-bearing mice.

|  | Median Survival (d) | Survival Rate (%) | *P* values | |
| --- | --- | --- | --- | --- |
|  |  |  | v.s. CTRL | v.s. CBDCA+α-PD-1 |
| CTRL | 53 | 0 |  | <0.0001 |
| α-PD-1 | 60 | 0 | n.s. | <0.0001 |
| CBDCA | 58 | 0 | n.s. | <0.0001 |
| CBDCA+α-PD-1 | 110 | 50 | <0.0001 |  |

Note: Survivals were analyzed by the Kaplan-Meier method and compared by the log-rank test. n.s., no significance. The same experiments as Figure 3.

Supplemental Table 12: Survival of secondary E0771 tumor-bearing mice.

|  | Median Survival (d) | Survival Rate (%) | *P* values | |
| --- | --- | --- | --- | --- |
|  |  |  | v.s. CTRL | v.s. CBDCA+α-PD-1 |
| CTRL | 58 | 0 |  | <0.0001 |
| α-PD-1 | 69 | 0 | n.s. | <0.0001 |
| CBDCA | 62 | 0 | n.s. | <0.0001 |
| CBDCA+α-PD-1 | Not Reached | 70 | <0.0001 |  |

Note: Survivals were analyzed by the Kaplan-Meier method and compared by the log-rank test. n.s., no significance. The same experiments as Figure 3.
